# Trace elements in dried blood spots as potential discriminating features for metabolic disorder diagnosis in newborns

**DOI:** 10.1093/mtomcs/mfab018

**Published:** 2021-04-30

**Authors:** Jorge Moreda-Piñeiro, José A Cocho, María Luz Couce, Antonio Moreda-Piñeiro, Pilar Bermejo-Barrera

**Affiliations:** Department of Chemistry, Faculty of Sciences, Universidade da Coruña, Grupo Química Analítica Aplicada (QANAP), University Institute of Research in Environmental Studies (IUMA), Centro de Investigaciones Científicas Avanzadas (CICA), Campus de A Coruña, s/n, 15071 A Coruña, Spain; Unit of Diagnosis and Treatment of Congenital Metabolic Diseases, Department of Pediatrics, University Hospital of Santiago de Compostela, IDIS, CIBERER, A Choupana, s/n, 15706 Santiago de Compostela, Spain; Unit of Diagnosis and Treatment of Congenital Metabolic Diseases, Department of Pediatrics, University Hospital of Santiago de Compostela, IDIS, CIBERER, A Choupana, s/n, 15706 Santiago de Compostela, Spain; Group of Trace Elements, Speciation and Spectroscopy (GETEE), Strategic Grouping in Materials (AEMAT), Department of Analytical Chemistry, Nutrition and Bromatology, Faculty of Chemistry, Universidade de Santiago de Compostela, Avenida das Ciencias, s/n, 15782 Santiago de Compostela, Spain; Group of Trace Elements, Speciation and Spectroscopy (GETEE), Strategic Grouping in Materials (AEMAT), Department of Analytical Chemistry, Nutrition and Bromatology, Faculty of Chemistry, Universidade de Santiago de Compostela, Avenida das Ciencias, s/n, 15782 Santiago de Compostela, Spain

**Keywords:** classification, metabolic disorders, dried blood spot, newborns, multi-element determinations, pattern recognition techniques

## Abstract

Trace elements in dried blood spots (DBSs) from newborns were determined by laser ablation coupled with inductively coupled plasma mass spectrometry, and data were subjected to chemometric evaluation in an attempt to classify healthy newborns and newborns suffering from metabolic disorders. Unsupervised [principal component analysis (PCA) and cluster analysis (CA)] and supervised [linear discriminant analysis (LDA) and soft independent modeling by class analogy (SIMCA)] pattern recognition techniques were used as classification techniques. PCA and CA have shown a clear tendency to form two groups (healthy newborns and newborns suffering from metabolic disorders). LDA and SIMCA have predicted that 90.5% and 83.9% of originally grouped healthy newborn cases were correctly classified by LDA and SIMCA, respectively. In addition, these percentages were 97.6% (LDA) and 80.6% (SIMCA) for DBSs from newborns suffering from metabolic disorders. However, SIMCA has only detected one misclassified DBS from the healthy group, and the lower percentage is attributed to four DBSs from the healthy newborn group and five DBSs from newborns with disorders that were found as belonging to both categories (healthy newborns and newborns with disorders) in the training set. LDA also gave a percentage of grouped maple syrup urine disease (MSUD) cases correctly classified of 100%, although the percentage fells to 66.7% when classifying phenylketonuria (PKU) cases. Finally, essential elements such as Fe, K, Rb, and Zn were found to be matched (correlated) with the concentration of amino acids such as phenylalanine, valine, and leucine, biomarkers linked with MSUD and PKU diseases.

## Graphical Abstract

**Graphical Abstract:**
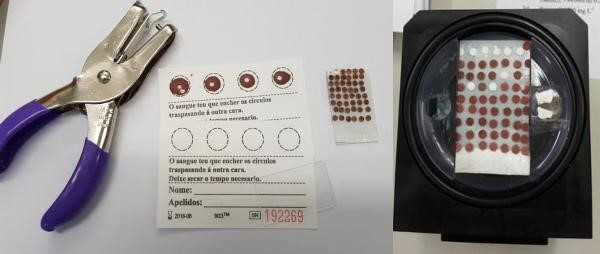
New-born screening programs, mainly based on dried blood spots (DBS), allow the early identification and diagnosis of several diseases regarding to metabolic disorders. Each specific disease is detected by assessing a certain bio-compound or a group of bio-compounds, by well-established HPLC-MS/MS methodologies. However, the changes on trace elements levels in blood when a metabolic disease is present could help to an easy diagnosis. Since the availability of DBS samples and the small amount of sample required for LA-ICP-MS, the multi-element determination in DBSs from new-born could be a complementary tool in new-born screening programs.

## Introduction

The diagnosis and early treatment of multiple congenital, metabolic–endocrine, and hereditary disorders through newborn screening (NBS) programs have been steadily increasing and they will continue to expand as the natural histories of additional disorders are understood and new treatment options are developed.^1^ NBS testing methods detect newborns’ disorders by assessing biomolecules such as proteins, enzymes, hormones, and fatty acids in newborns’ blood. Physiological indicators such as acylcarnitines and amino acids are used for diagnosing inherited disorders of amino acids and fatty acids in newborns and children,^2,3^ and these biomarkers are commonly quantified in dried blood spots (DBSs).^4^ The DBS technique offers several advantages over other sample pretreatment methods, such as DBS is a simple and inexpensive methodology (pricking the heel with a lancet is easy and DBS collection can be performed by relatively untrained personnel); DBS requires a minimal sample volume; DBS offers a great sample stability (degradation processes of analytes are slowed down), and the samples are easy to store and transport.^5–11^

Oligoelements and metals are being used as alternatives to biomolecules for detecting metabolic–endocrine, hereditary, and neurodegenerative diseases.^7–9^ In addition, metal profiles in serum could be used to study the progression of these diseases.^10^ Oligoelements are essential for life since they maintain the normal functions of many biochemical and physiological processes. However, to the best of our knowledge, a systematic study involving several oligoelements/metals and metabolic disorders in newborns has not been performed.

In the current research, a novel study based on oligoelement and metal data in DBSs from newborns for discriminating between healthy newborns and newborns suffering from metabolic disorders has been investigated. Diseases such as aminoacidopathies and organic acidemias (maple syrup urine disease, MSUD; phenylketonuria, PKU; tyrosinemia, Tyr; citrullinemia type I, CIT; hydroxymethyl glutaric aciduria, HMG; non-ketotic hyperglycinemia, NKH; methylmalonic acidemia, MUT; homocystinuria, HCY; and 3-methylcrotonyl-CoA carboxylase deficiency, 3-MCCD) and fatty acid oxidation disorders (medium-chain acyl-CoA dehydrogenase deficiency, MCAD; long-chain 3-hydroxyacyl-CoA dehydrogenase deficiency, LCHAD; and very long-chain 3-hydroxyacyl-CoA dehydrogenase deficiency, VLCAD) have been included in the study. Laser ablation (LA) coupled with inductively coupled plasma mass spectrometry (ICP-MS) has been successfully applied for the direct analysis of several elements in biological samples and DBSs.^11^ The use of LA avoids time-consuming pretreatments, and also prevents contamination of facilities during the extraction process.^11,12^ Pattern recognition techniques such as principal component analysis (PCA), cluster analysis (CA), linear discriminant analysis (LDA), and soft independent modeling by class analogy (SIMCA) have been used for visualizing groups of DBSs from healthy newborns and newborns suffering from metabolic disorders. A subclassification of DBSs into the newborns suffering from MSUD and PKU has also been attempted. Finally, relationships between oligoelements and some aminoacidopathies have also been studied.

## Materials and methods

### Instrumentation

A high-resolution magnetic sector-based ICP-MS (ELEMENT XR, Thermo Finnigan, Thermo Instruments, Bremen, Germany) coupled to an LA system (UP213, New Wave Research, Inc., St Neots, Cambridgeshire, UK) and a Nikon microscope head were used for the multi-element quantification in DBSs. LA of DBSs was performed using a focused high-performance Nd:YAG laser beam in the line scanning ablation mode: a wavelength of 213 nm, a laser fluency of 3.0 J cm^–2^, a repetition rate of 20 Hz, a scanning speed of 8.0 µm s^–1^, an ablation depth of 0 µm, and a laser spot diameter of 90 mm (Table S1). The ablated material was transported by He gas (1.0 ml min^–1^) into the ICP. The experimental parameters of LA-ICP-MS were optimized at the beginning of each analysis set by ablating an NIST 612 glass standard (National Institute of Standards and Technology, Gaithersburg, MD, USA) in accordance with the operating conditions listed in Table S1. A wash-up time of 55 s was inserted between successive ablations to minimize or avoid memory effects. Elements in DBSs were measured in low-resolution mode.

### Reagents and materials

Ultrapure water with a resistance of 18 MΩ cm was obtained from a Milli-Q^®^ purification device (Millipore Corporation, Bedford, MA, USA). Multi-element standard solutions were prepared by combining stock standard solutions of 1.000 g l^−1^ (Cu, Fe, P, Pb, Rb, Sb, Sr, and Zn) and 5.000 g l^−1^ (Ca, K, Mg, and Na) supplied by Merck (Poole, Dorset, UK). Seronorm^™^ Trace Elements Human Whole Blood level II and level III were obtained from Sero (Billingstad, Norway). Certified reference materials (CRMs) were supplied as a lyophilized material and they were reconstituted in ultrapure water. NIST 612 glass standard was used for LA-ICP-MS instrumental condition optimization and calibration. Whatman 903 paper cards were obtained from Whatman (Dassel, Germany). The steel punching tool was from McGill Inc. (Jacksonville, FL, USA).

### DBS collection and storage

Eighty-four anonymized whole blood samples impregnated on Whatman 903 paper (42 DBS samples from healthy newborns and 42 samples from newborns with metabolic disorders) were supplied by the Unit of Diagnosis and Treatment of Congenital Metabolic Diseases, Department of Pediatrics, University Hospital of Santiago de Compostela (Santiago de Compostela, Spain). Each DBS (stored at −20°C since their sampling and analysis in the Laboratory of Metabolic and Nutritional Disorders) consists of four spots from drops of whole blood obtained from a newborn’s heel by using a single-use safety lancet.^13^ Filter papers containing the anonymized dried blood specimens were protected (use of a sturdy paper overlay supplied by Whatman) and stored in the desiccator at constant temperature (20 ± 1°C) and controlled relative humidity (50 ± 5%). Relevant data on DBS samples (newborns’ weight, days of life before sample collection, and metabolic disease detected) are summarized in Table S2. Two aminoacidopathies, PKU and MSUD, were diagnosed in 18 and 10 newborns, respectively. Other aminoacidopathies and organic acidemias (Tyr, CIT, HMG, NKH, 3-MCCD, HCY, and MUT), and fatty acid oxidation disorders (MCAD, LCHAD, and VLCAD) were only diagnosed in few newborns. Concentrations and ratios of several amino acids, fatty acids, and their metabolites,^14,15^ linked with these disorders, are also enclosed in Table S2.

### DBS preparation

**Fig. 1:**
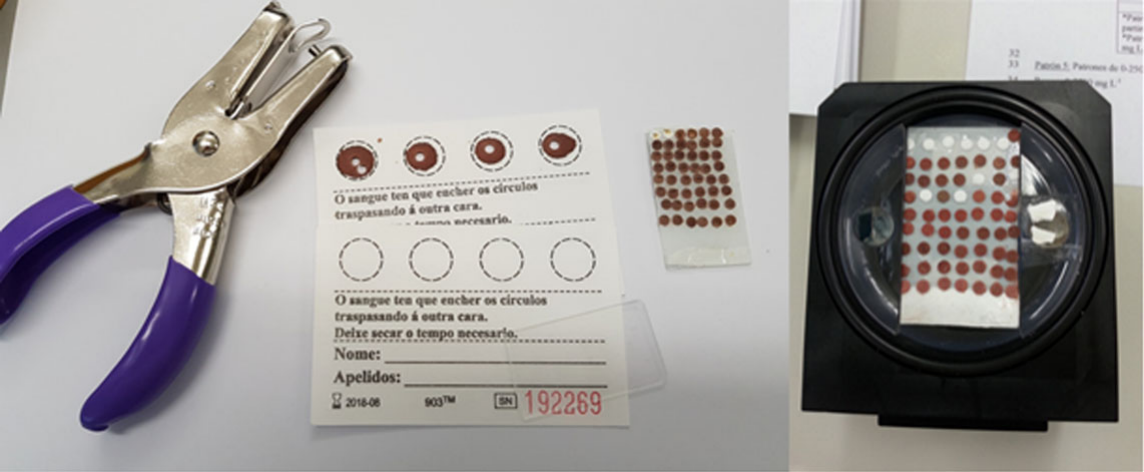
Circular filter paper disks (3 mm) with DBSs from different real samples and reconstituted Seronorm CRMs.

Circular disks of 3 mm diameter were cut from DBS samples using a steel punching tool in an ultraclean vertical laminar air flow hood NU-156 (NUAIRE, Plymouth, MA, USA). These precut disks were applied to glass microscopic slides using double-sided tape (Fig. 1). Seronorm^™^ level II and level III (calibration and accuracy assessment) were reconstituted with ultrapure water in an ultraclean vertical laminar air flow hood, and 20 µl aliquots were pipetted and spotted onto the paper cards. After air-drying (ultraclean vertical laminar air flow hood), DBSs were subjected to the procedure described above (3 mm diameter circular disks and fixing onto glass microscopic slides).

### Procedure for simultaneous multi-elemental analysis of DBSs

Multi-element determinations in DBS samples (instrumental operating conditions in Table S1) were performed by two-point calibration using DBSs prepared from Seronorm^™^ level III. ^13^C was used as an internal standard to correct sample-to-sample variations in the ion signal intensity during the ablation step. Carbon in human blood presents a homogeneous distribution and provides constant concentration. Similarly, carbon is also homogeneously present in the Whatman cards and the ^13^C-based correction is therefore attributed to ^13^C from paper cards and blood (approximately a 44% of ^13^C is attributed to the paper card). Ca, Cu, Fe, K, Mg, Na, P, Pb, Rb, Sb, Sr, and Zn concentrations (*N* = 3), calculated from average ion intensities, and data reduction were carried out with the commercial GLITTER^®^ software package (New Wave Research, Inc., St Neots, Cambridgeshire, UK). Relevant elements such as iodine and manganese, linked to several disorders, were not considered since the levels in DBSs were found to be lower than the LOQs.^13^ Two-point calibration strategy (a blank card as a first point, and Seronorm III-high level as a second point, measured in triplicate) was performed by GLITTER^®^ software package. After blank and Seronorm III measurements, Seronorm II (accuracy check) and five DBS samples were measured also in triplicate. Then, a new calibration was done, and other Seronorm II DBS (accuracy check) and five DBS samples were measured in triplicate. The same sequence is repeated depending on the number of DBS samples analyzed in each analysis section. GLITTER^®^ is a data reduction software for LA microprobe and provides simpler, more consistent, and faster reduction of LA-ICP-MS data. GLITTER software allows concentrations to be calculated as soon as standards and few samples have been analyzed, and data are instantaneously adjusted to take into account changes in the instrumental drift.

DBSs of Seronorm^™^ level II were used as standard reference material to validate the accuracy of the procedure. Two-point calibration using DBS of Seronorm^™^ level III and ^13^C as internal standard offers results that are in good agreement with the declared values when Seronorm^™^ level II was analyzed. Found concentrations (14.5 ± 1.2, 1.4 ± 0.11, 356 ± 15.1, 1078 ± 70.9, 14.6 ± 0.48, 1514 ± 87.8, 186 ± 7.4, 0.35 ± 0.025, 1.39 ± 0.11, 0.026 ± 0.008, 0.033 ± 0.007, and 7.2 ± 0.13 mg l^−^^1^ for Ca, Cu, Fe, K, Mg, Na, P, Pb, Sb, Sr, Rb, and Zn, respectively) were in good agreement with the certified/informative values (15.0, 1.34 ± 0.27, 349, 1100, 14.4, 1543, 183, 0.34 ± 0.068, 1.41, 0.026 ± 0.005, 0.036, and 7.1 ± 1.4 mg l^−1^ for Ca, Cu, Fe, K, Mg, Na, P, Pb, Sb, Sr, Rb, and Zn, respectively). These findings have been verified by applying the *t*-test (95% confidence level, 20 degrees of freedom) since *t*_cal_ values (1.85, 1.96, 1.60, 1.40, 1.61, 1.47, 1.61, 1.91, 1.26, 1.61, 1.62, and 1.76 for Ca, Cu, Fe, K, Mg, Na, P, Pb, Sb, Sr, Rb, and Zn, respectively) were found to be lower than the *t*_tab_ value of 2.09. Elements in DBSs can therefore be directly assessed with two-point calibration technique to ensure the accuracy of analysis.

The LOQs (concentration providing a signal equivalent to 10 times the noise from 11 measurements of a blank filter) were 13.1, 0.60, 40.0, 120, 11.9, 105, 152, 0.60, and 1.5 mg l^−^^1^ for Ca, Cu, Fe, K, Mg, Na, P, Rb, and Zn, respectively; and 7.0, 1.6, and 3.6 µg l^−^^1^ for Pb, Sb, and Sr, respectively. The obtained LOQ values were low enough to perform multi-element determination in DBSs. The repeatability of the procedure was tested by ablating 20 blood areas of the same DBS prepared from a 20 µl drop of reconstituted Seronorm™ level II, and the RSD values were lower than 12% for all elements. Finally, the reproducibility of the procedure by ablating seven DBSs (20 µl drop of reconstituted Seronorm™ level II each) and by analyzing each DBS twice was lower than 17% for all elements, which implies good repeatability of DBS preparation and spotting.

### Statistical treatment of data

Univariate analysis, correlation analysis, PCA, CA, and LDA were performed with Statgraphics Centurion XVII-X64 routine (Statgraphics Corporation, The Plains, VA, USA), whereas SIMCA was performed with THE UNSCRAMBLER 7.01 (CAMO ASA, Trondheim, Norway). The standardized skewness and standardized kurtosis statistics for normal distribution assessment of elemental concentrations were also performed with Statgraphics Centurion XVII-X64 routine.

## Results and discussion

### Element concentrations in DBSs

The concentrations of major elements Ca, Cu, Fe, K, Mg, Na, P, Rb, and Zn in the DBSs (*N* = 84) under study varied in the range of <13.1–35.7, <0.60–8.5, 363.4–1416, 1583–5711, 2.4–419.0, 1373–3920, 260.1–793.2, <0.60–6.3, and 1.6–13.1 mg l^−1^, respectively. For minor elements, the concentration ranges were as follows: <1.6–169.5 µg l^−1^ (Sb), <3.6–293.0 µg l^−1^ (Sr), and <7.0–226.9 µg l^−1^ (Pb) (Table S3). Average element concentrations in 84 DBSs as well as other statistics (RSD, maximum and minimum concentration, and range) are summarized in Table 1. Average element concentrations and statistics in DBSs from healthy newborns (*N* = 42) and newborns with metabolic disorders (*N* = 42) are also listed in Table 1. Elements such as Mg, Pb, Sb, and Sr have shown high variation among DBS samples, with RSDs higher than 117% (Table 1). Otherwise, Ca, Fe, K, Na, and P offer RSDs lower than 30%. The same patterns were observed for healthy newborns’ DBSs and DBSs from newborns suffering from metabolic disorders (Table 1). The standardized skewness and standardized kurtosis statistics were used to determine whether elemental concentrations for the different groups come from a normal distribution. Values of these statistics outside the range of −2 to +2 indicate significant departures from normality. As can be seen, Cu, K, Na, P, Rb, and Sr concentrations for healthy newborns, and Ca, Fe, K, Na, P, and Rb concentrations for newborns suffering from metabolic disorders show normal distributions (Table 1).

**Table 1.** Element concentrations expressed as mg l^−1^ in several DBSs from newborns along with statistical results

	Average	Median	RSD (%)	25th percentile	75th percentile	Range	Skewness value	Kurtosis value
All newborns (84 DBSs)								
Ca	16.8	13.1	30.7	13.1	20.6	22.6	5.8	4.6
Cu	1.2	1.1	73.1	0.87	1.3	7.9	24.4	94.8
Fe	703.7	675.3	28.2	570.0	822.9	1053	3.8	2.8
K	3023	2943	29.7	2302	3699	4128	2.5	0.40
Mg	59.3	31.1	117.4	24.1	71.4	416.6	10.4	17.2
Na	2511	2506	19.9	2186	2811	2547	0.66	0.66
P	491.6	478.3	21.7	417.1	549.6	533.1	1.5	0.55
Pb	27.0^a^	17.9^a^	126.6^a^	7.0^a^	30.2^a^	219.9^a^	14.3	32.5
Rb	2.8	3.0	48.9	1.5	3.7	5.7	0.26	−1.4
Sb	10.3^a^	3.1^a^	228.7^a^	1.6^a^	6.9^a^	167.9^a^	18.9	54.8
Sr	17.5^a^	15.4^a^	179.0^a^	8.4^a^	18.5^a^	289.4^a^	31.2	138.4
Zn	4.8	4.7	47.0	3.1	5.9	11.5	3.9	2.8
Healthy newborns (42 DBSs)								
Ca	13.2	13.1	3.5	13.1	13.1	2.3	11.8	25.4
Cu	0.93	0.89	18.4	0.81	1.0	0.70	1.9	−0.13
Fe	845.5	825.3	21.2	732.1	888.4	896.4	3.1	2.6
K	3697	3726	20.7	3317	3922	4126	0.84	1.9
Mg	78.2	64.4	103.2	21.0	90.0	416.6	5.7	7.7
Na	2380	2446	20.6	2091	2715	1895	−0.73	−0.57
P	544.6	538	19.6	461.4	658.9	421.4	1.1	−0.99
Pb	19.9^a^	8.8^a^	180.0^a^	7.0^a^	16.6^a^	219.9^a^	14.3	42.3
Rb	3.8	3.7	26.1	3.3	4.5	5.4	−0.66	1.5
Sb	8.7^a^	4.4^a^	132.4^a^	1.6^a^	9.5^a^	43.2^a^	6.0	5.9
Sr	12.0^a^	12.9^a^	51.8^a^	6.2^a^	17.2^a^	18.6^a^	−0.16	−1.8
Zn	3.4	3.0	50.8	2.3	3.7	9.4	5.9	7.6
Newborns suffering from metabolic disorders (42 DBSs)								
Ca	20.0	20.4	26.6	16.0	22.7	22.6	1.5	1.9
Cu	1.5	1.2	78.2	1.1	1.4	7.8	13.2	37.7
Fe	574.9	576.5	18.4	491.4	630.4	481.7	0.50	−0.28
K	2411	2327	19.2	2120	2602	1915	1.9	0.33
Mg	42.2	28.7	126.0	24.8	32.8	246.1	11.4	24.4
Na	2630	2534	18.5	2374	2894	2178	1.8	0.66
P	443.3	464.8	18.4	389.7	500.0	339.5	−1.5	−0.019
Pb	33.4^a^	25.0^a^	94.6^a^	17.7^a^	37.3^a^	152.5^a^	7.4	11.7
Rb	1.9	1.6	53.8	1.1	2.7	4.0	1.5	−0.75
Sb	11.7^a^	2.4^a^	261.5^a^	1.6^a^	6.3^a^	167.9^a^	10.9	23.7
Sr	22.6^a^	16.7^a^	188.4^a^	11.9^a^	19.7^a^	289.4^a^	16.1	51.1
Zn	6.1	5.7	31.1	5.0	6.8	9.8	4.2	5.7

^a^Values expressed as µg l^−1^.

Cu, Fe, Mg, and Zn concentrations (Table 1) in healthy newborns are in good agreement with reported data in neonates and children (under 1 year of age) blood (0.99–1.09, 413.3–450.9, 3.35–5.4, and 37.0 ± 3.9 mg l^−1^ for Cu, Fe, Mg, and Zn, respectively).^16,17^ Ca levels (Table 1) are lower than reported values for children aged 0–14 years old (74.2 ± 7.21 mg l^−1^).^17^ The concentrations of non-essential elements, such as Pb, are in good agreement with reported values from newborns and 0–7 years old children (<10–300 µg l^−1^).^5,16,18–20^ As can be seen in Table 1, Cu (0.66–8.5 mg l^−1^) and Mg (8.8–254.9 mg l^−1^) levels in newborns suffering from metabolic disorders are considerably higher than reported Cu and Mg values.

A first attempt to find tendencies has consisted of comparing the averages and SDs of elements from two groups (healthy and/or with metabolic disorders) that show normal distributions. Table 2 shows statistically significant differences (95.0% confidence level) between DBS samples from both groups (the *P*-values of the F-test is lower than 0.05) for K, Na, P, and Rb. These findings suggest that the concentration of some essential elements in newborn blood could be used to detect metabolic diseases.

**Table 2.** Analysis of variance of target elements for several DBSs from newborns with and without metabolic disorders

		Sum of squares	Degrees of freedom	Mean square	F-ratio	*P*-value
K	Between groups	3.5 × 10^13^	1	3.5 × 10^13^	89.5	0.0000
	Within groups	3.2 × 10^13^	82	3.9 × 10^11^		
Na	Between groups	1.3 × 10^12^	1	1.3 × 10^12^	5.4	0.0210
	Within groups	2.0 × 10^13^	82	2.4 × 10^11^		
P	Between groups	2.1 × 10^11^	1	2.1 × 10^11^	24.2	0.0000
	Within groups	7.3 × 10^11^	82	8.9 × 10^9^		
Rb	Between groups	7.3 × 10^7^	1	7.3 × 10^7^	70.4	0.0000
	Within groups	8.5 × 10^7^	82	1.0 × 10^6^		

### Data analysis

Elements in DBSs from newborns with or without metabolic disorders (Table 1) will be explored using univariate approaches and unsupervised recognition techniques such as PCA and CA. The DBS samples will also be classified using supervised recognition approaches (LDA and SIMCA). In order to find the elements that could be matched with most of diseases (MSUD and PKU) diagnosed in newborn samples, several amino acids and fatty acids linked with those aminoacidopathy disorders were also included and studied in combination with the assessed elements (PCA and CA). Amino acids and fatty acids concentrations (Table S2) were provided by the Unit of Diagnosis and Treatment of Congenital Metabolic Diseases, Department of Pediatrics, University Hospital of Santiago de Compostela. The data are part of the set of parameters included in the NBS program of the Autonomous Region of Galicia (Spain).

*Univariate analysis of elements:* A statistic based on matrix correlation (Pearson product moment correlations between each pair of variables) was performed between the target elements, amino acids, and fatty acids concentrations in DBS samples (Table S4). Results showed from a moderate to a strong correlation among certain target elements (*P*-values lower than 0.05 at 95% confidence interval): Ca was found to be correlated with Cu (*P*-value = 0.0007), Fe (*P*-value = 0.0000), K (*P*-value = 0.0000), Mg (*P*-value = 0.0282), Na (*P*-value = 0.0004), Pb (*P*-value = 0.0025), Rb (*P*-value = 0.0000), Sb (*P*-value = 0.0261), Sr (*P*-value = 0.0012), and Zn (*P*-value = 0.0005). Cu was correlated with Na (*P*-value = 0.0018) and Zn (*P*-value = 0.0049); Fe was correlated with K (*P*-value = 0.0000), Mg (*P*-value = 0.0002), P (*P*-value = 0.0002), and Rb (*P*-value = 0.0002). K showed correlation with Mg (*P*-value = 0.0185), P (*P*-value = 0.0001), Rb (*P*-value = 0.0000), Sr (*P*-value = 0.0482), and Zn (*P*-value = 0.0030), whereas Mg was found to be correlated with Rb (*P*-value = 0.0035). Finally, significant correlation was found between Na and Zn (*P*-value = 0.0155), between P and Rb (*P*-value = 0.0055), between P and Zn (*P*-value = 0.0037), between Sr with Rb (*P*-value = 0.0213), and between Rb with Zn (*P*-value = 0.0024). The relationships between the elements appear complex and difficult to explain individually. Ca was found to be correlated with all elements, except P, whereas Pb was found to be correlated only with Ca (positive correlation). Further elucidations may be obtained using more powerful chemometric techniques such as PCA and CA.

A statistic based on matrix correlation was also performed between the target elements, amino acids, and fatty acids concentrations in DBS samples (Table S4). Results showed from a moderate to a strong correlation among certain amino acids/fatty acids and elements (*P*-values lower than 0.05 at 95% confidence interval): hexanoyl was correlated with Ca (*P*-value = 0.0021) and Pb (*P*-value = 0.0028), whereas octanoyl was correlated with Ca (*P*-value = 0.0000), Na (*P*-value = 0.0115), Pb (*P*-value = 0.0000), Sb (*P*-value = 0.0015), and Zn (*P*-value = 0.0496). Significant correlations were also observed between glycine and Cu (*P*-value = 0.0286), Fe (*P*-value = 0.0133), K (*P*-value = 0.0095), Na (*P*-value = 0.0071), P (*P*-value = 0.0056), Rb (*P*-value = 0.0492), and Zn (*P*-value = 0.0003). Finally, citrulline was found to be correlated with Ca (*P*-value = 0.0418), Fe (*P*-value = 0.0278), K (*P*-value = 0.0269), and P (*P*-value = 0.0053). The relationships between the elements and valine concentration and phenylalanine/tyrosine ratio (linked with MSUD and PKU, respectively) were also studied. Correlation matrix (Table S4) shows a positive correlation between valine content and Ca and Zn concentrations (*P*-values of 0.0001 and 0.0065 at 95% confidence interval for Ca and Zn, respectively), whereas a negative correlation was found for Fe, K, and Rb contents (*P*-values of 0.0497, 0.0033, and 0.0076 at 95% confidence interval for Fe, K, and Rb, respectively). Correlation matrix also shows a negative correlation between phenylalanine/tyrosine ratio and Fe and K contents (*P*-values of 0.0362 and 0.0017 at 95% confidence interval for Fe and K, respectively). Finally, a positive correlation was achieved for phenylalanine/tyrosine ratio and Zn contents (*P*-value of 0.0053 at 95% confidence interval).

**Fig. 2:**
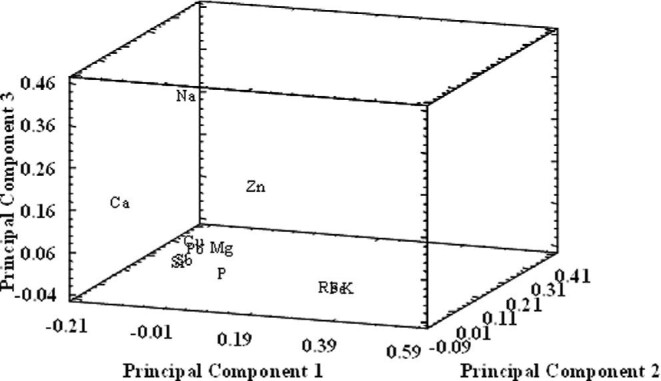
3D plot of factor loadings for 12 target elements.

*Principal component analysis and cluster analysis:* PCA has been attempted with a dataset in which Ca, Cu, Fe, K, Mg, Na, P, Pb, Rb, Sb, Sr, and Zn concentrations in DBSs were the discriminating variables, and 84 DBSs were the objects. PCA was performed after dataset homogenization (half-range and central value transformation),^21^ cross-validation and normalization (Varimax rotation). Three principal components have been found to explain 66.9% of the total variance of the data. PC1 (41.4%) and PC2 (14.0%) represented 55.4% of variance, while PC3 captured 11.5% of variance. Results show large loadings for K (+0.412), Rb (+0.377), Fe (+0.349), and Ca (−0.210) on the first component, PC1 (Table 3; Fig. 2). The second PC offers the highest weights for Zn (+0.374) content, whereas the third PC offers the highest weights for Na (+0.377). These findings agree with those obtained after univariate analysis, which showed a strong correlation between Fe, K, and Zn contents with valine concentration (amino acid related to MSUD), and also with the phenylalanine content and with the phenylalanine/tyrosine ratio, parameters linked to PKU.

**Table 3.** Factor loadings for elements, after a normalized Varimax rotation for the first three principal components

	Loadings		
	PC1	PC2	PC3
Na	−0.03231	+0.04322	+0.3775
Mg	+0.07581	+0.0008784	+0.04069
P	+0.1122	−0.05501	+0.001388
K	+0.4121	−0.06277	−0.01065
Ca	−0.2099	+0.07860	+0.09837
Fe	+0.3490	+0.001588	−0.03645
Cu	−0.006707	+0.03029	+0.03622
Zn	−0.06101	+0.3742	+0.04430
Rb	+0.3774	−0.08134	−0.0006126
Sr	−0.02813	+0.001445	−0.003068
Sb	−0.007340	−0.004403	+0.007462
Pb	+0.0002377	+0.02193	+0.02289

In addition, Ca has been reported to be involved in the effects of α-ketoisocaproic acid (the main accumulated metabolite in MSUD), and in the phosphorylating system associated with intermediate filament proteins.^22^

**Fig. 3:**
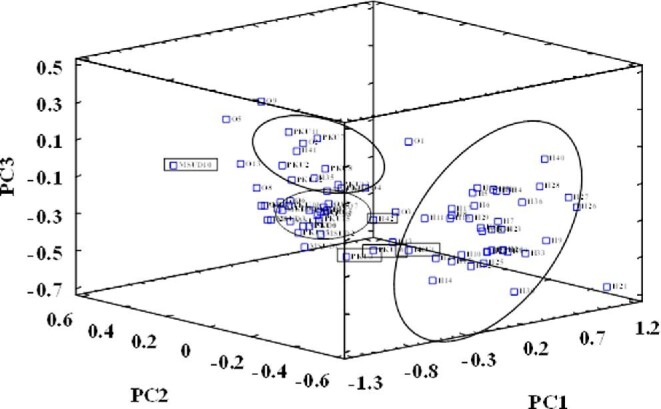
3D plots of scores for 84 DBS samples: healthy newborns (H) and newborns with metabolic disorders (PUK, MSUD, and O for phenylketonuria, maple syrup urine disease, and others diseases, respectively).

Based on 12 element concentrations as discriminating features, DBSs were classified into two groups (3D PC score graphs; Fig. 3). DBSs from healthy newborns were grouped at the right side in Fig. 3 (H, healthy). DBSs from newborns suffering from metabolic disorders were found to be grouped at the left side in Fig. 3: MSUD cases form a compact group at the bottom-left part of the plot and PKU cases are mainly grouped at the upper-left part of the graph, quite close to the compact MSUD group. DBS cases from other metabolic diseases do not form a grouping, and they were found to be dispersed and close to the MSUD and PKU groups. Three of the four MCDA cases (samples coded as O5, O9, and O13) were found to be close, and other samples from patients suffering from HCY (O11), HMG (O7), MCCD (O12), LCHAD (O6), MUT (O2), NKH (O8), Tyr (O10), and VLCAD (O4) appear to be dispersed into the main PKU and MSUD groups. Although, element data contain significant information to achieve a satisfactory separation between healthy newborns and newborns suffering from metabolic diseases, there have been found some misleading groupings, such as samples from healthy newborns (H35, H37, H41, and H42) that were found to be grouped with samples belonging to newborn patients, and a sample from a newborn patient suffering from PKU (PKU1) that was found to be mixed with samples belonging to healthy newborns.

**Fig. 4:**
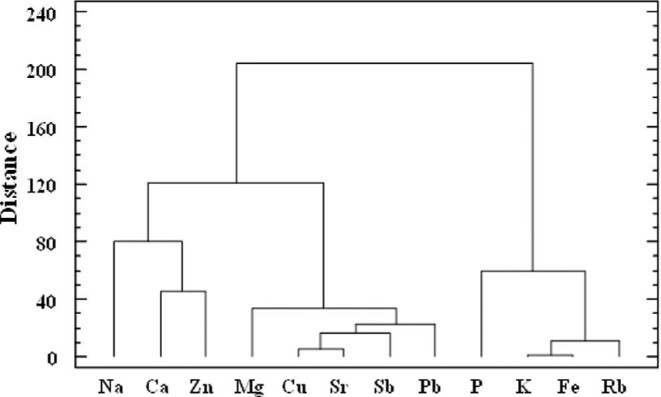
Dendrogram of cluster analysis for 12 target elements.

**Fig. 5:**
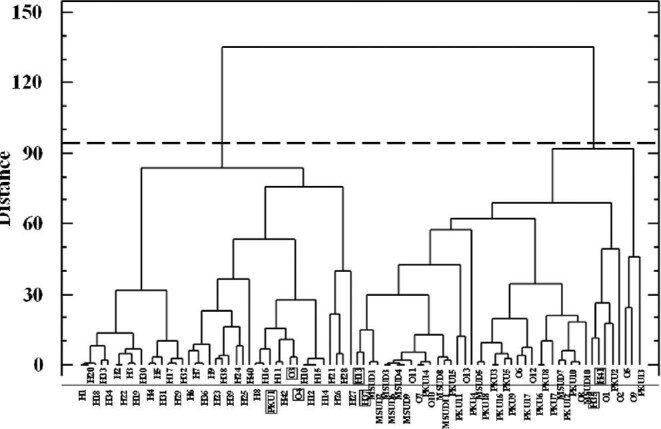
Dendrogram of cluster analysis of 84 DBS samples: healthy newborns (H) and newborns with metabolic disorders (PUK, MSUD, and O for phenylketonuria, maple syrup urine disease, and others diseases, respectively).

CA was performed after half-range and central value transformation, using the Ward's method and the squared Euclidian distance for similarity. Results from CA (Figs 4 and 5) agreed to those offered by PCA. Regarding discriminating variables, three clusters were observed at a distance of 90. Beginning from the right (Fig. 4), the first cluster is formed by the discriminating variables P, K, Fe, and Rb; most of them (K, Fe, and Rb) were found to explain PC1 after PCA and were also found to be correlated with valine concentration (amino acid related to MSUD) and with the phenylalanine content and with phenylalanine/tyrosine ratio (parameters used for PKU diagnosis). The second cluster is formed by Mg, Cu, Sr, Sb, and Pb concentrations, variables that were found to give poor discrimination in PCA. Finally, the third cluster was formed by Na (feature that explains PC3 in PCA), Ca, and Zn (variable that is the main component in PC2 in PCA, and that was found to be correlated with MSDU and PKU markers).

Regarding DBS samples, two big clusters (healthy newborns and newborns suffering from metabolic disorders) were observed as shown in dendrogram (Fig. 5) at a linkage distance of 95 (dashed line in Fig. 5). The first cluster (right) is mainly formed by samples from newborn patients (39 samples), although 4 samples from healthy newborns have been grouped in this cluster (samples coded as H41, H35, H37, and H13). Samples H41, H35, and H37 were also found as misclassified after PCA. The second cluster (left) is formed by 41 objects, from which 38 samples were from healthy newborns. Three misclassified samples from newborns suffering from PKU (PKU1), Citr I (O3), and VLCAD (O4) were found to belong to this second cluster. Samples PKU1 and O3 (PKU and Citr I, respectively) were also found as misclassified samples (samples close to the group formed by samples from healthy newborns) after PCA.

*Linear discriminant analysis:* LDA, a supervised pattern recognition method, allows the DBSs’ (objects) classification into groups (two groups or classes) with predetermined models for the classes: healthy newborns (Group H) and newborns suffering from metabolic disorders (Group D). The recognition ability for the two groups was highly satisfactory (Table 4), and the percentage of correctly classified cases was 94.05%. Four DBSs from healthy newborns (H35, H37, H42, and H43) were misclassified as a sample of Group I (newborns with metabolic disorders) leading to a percentage of correctly classified cases of 90.48%. One DBS from newborn with metabolic disorders (O1) was also misclassified as healthy newborn (Group H), implying a 97.62% as the percentage of DBSs correctly classified. Regarding misclassified cases from healthy newborn group (H35, H37, H42, and H43), LDA results agree with those observed after PCA, and these samples were grouped as belonging to newborns suffering from metabolic disorders. However, LDA results do not match with those offered by PCA and CA for samples from the newborns suffering from metabolic disorder group since misclassified sample O1 by LDA was found to be correctly grouped as belonging to the group formed by newborns suffering from metabolic disorders by PCA and CA. In addition, misleading samples PKU1 (PCA and CA results) and O3 and O4 (CA results) have been correctly classified by LDA.

**Table 4.** Classification of DBS samples results into two groups (healthy newborns and newborns suffering from metabolic disorders) after LDA

		Predicted group membership		
Actual group	No. of cases	% Classification	Group H (healthy)	Group D (metabolic disorders)
Group H (healthy)	42	90.48	38	4
Group D (metabolic disorders)	42	97.62	1	41
Total	84	94.05	39	45

The first standardized discriminating function is described as − 0.0980 [Na] − 0.2474 [Mg] − 0.3241 [P] + 0.01576 [K] + 0.5494 [Ca] − 0.4394 [Fe] + 0.1396 [Cu] + 0.5274 [Zn] − 0.2386 [Rb] − 0.08669 [Sr] − 0.004012 [Sb] + 0.08647 [Pb]. The relative magnitudes of the coefficients in the discriminant equation confirm that Ca (+0.5494), Zn (+0.5274), Fe (−0.4394), P (−0.3241), and Mg (−0.2474) are the elements that mainly discriminate between groups. Elements such as Fe, Zn, and Ca were previously found to be important variables when grouping samples from healthy newborns and newborns suffering from diseases by PCA and CA. These variables were also found to be statistically correlated with PKU and MSUD markers. As previously mentioned, Ca has been reported to be associated with α-ketoisocaproic acid (MSUD's metabolite).^22^ Special mention must be given to Mg content, which was found to be a discriminating feature by LDA, and which was not found to be an important variable when applying PCA and CA. The relationship between Mg content and MSUD could be explained from recent reports that have stated that Mg is a core element of phosphatase Mg^2+^/Mn^2+^-dependent 1K (PPM1K), which is an important metabolic regulator associated with MSUD and with type 2 diabetes, as well as with other cardiovascular and neurological diseases.^23^

LDA was also carried out to attempt a subclassification of DBSs within the newborns suffering metabolic disorders group. Four classes were therefore considered: Group H (healthy newborns, 42 cases), Group M (newborns suffering from MSUD, 11 cases), Group P (newborns suffering from PKU, 18 cases), and Group O (newborns suffering from other aminoacidopathy disorders, 13 cases). In general, the recognition ability for the four groups was not satisfactory since the percentage of grouped cases correctly classified was 82.14% (Table 5). However, the percentage of MSUD cases correctly classified was 100%, which means that this metabolic disorder can be successfully classified using the element’s content. Regarding PKU, the percentage of cases correctly classified fell to 66.67%, and 6 from the 18 PKU cases were misclassified (2 cases as MSUD and 4 cases as belonging to other metabolic disorders). Bad classification (percentage of 61.54%, 5 misclassified cases from 13 cases) was obtained for cases included under the heading “other metabolic disorders.” This finding could be expected because this group is formed for few samples related to quite different metabolic diseases. Finally, as previously obtained for healthy newborns, four cases were found as misclassified samples (two as belonging to MSUD group and two cases as belonging to PKU group), which account for a percentage of correctly classified cases of 90.48%.

**Table 5.** Classification of DBS samples results into four groups (healthy newborns and newborns suffering from MSUD, PKU, and other metabolic disorders) after LDA

		Predicted group membership				
Actual group	No. of cases	% Classification	Group H (healthy)	Group M (MSUD)	Group P (PKU)	Group O (other metabolic disorders)
Group H (healthy)	42	90.48	38	2	2	0
Group M (MSUD)	11	100	0	11	0	0
Group P (PKU)	18	66.67	0	2	12	4
Group O (other metabolic disorders)	13	61.54	1	2	2	8
Total	84	82.1	39	17	16	12

First discriminant function: 0.06068 [Na] + 0.2366 [Mg] + 0.3874 [P] − 0.05671 [K] − 0.6226 [Ca] + 0.4602 [Fe] − 0.1815 [Cu] − 0.4561 [Zn] + 0.1722 [Rb] + 0.1606 [Sr] + 0.006083 [Sb] − 0.08971 [Pb].

Second discriminant function: + 0.2184 [Na] − 0.1143 [Mg] − 0.3685 [P] − 0.5551 [K] + 0.6681 [Ca] + 0.3973 [Fe] + 0.1715 [Cu] − 0.2173 [Zn] + 1.110 [Rb] + 0.07819 [Sr] + 0.09159 [Sb] − 0.3596 [Pb].

Table 5 shows the two standardized discriminant functions in which Ca (−0.6226), Fe (+0.4602), Zn (−0.4561), and P (+0.3874) were the main elements in the first standardized discriminant function, and Rb (+1.11), Ca (+0.6681), K (−0.5551), and Fe (+0.3973) offered the highest weights along the second standardized discriminant function.

*Soft independent modeling by class analogy:* Samples (42 cases from healthy newborns and 42 cases from newborns with metabolic disorders) were randomly divided into a training set (75% of samples) and a testing (prediction) set (25% of samples). The training set was formed by 31 samples from healthy newborns and 31 samples from newborns with metabolic disorders. The testing set was formed by 11 samples from healthy newborns and 11 cases from newborns with metabolic disorders. The latter set includes four PKU cases, three MSUD cases, two MCAD cases, one VLCAD case, and one Tyr case.

Results from SIMCA for training and prediction sets are listed in Table 6 for a significance level of 5%. Recognition percentages (training set) of samples correctly classified were found to be 83.87% and 80.65% for healthy newborns and newborns suffering from metabolic disorders, respectively. Four healthy newborn samples were identified as belonging to both healthy newborns and newborns suffering from metabolic disorders, whereas only one sample was misclassified. Regarding newborns suffering from metabolic disorders, five samples were also found to belong to both healthy newborns and newborns suffering from metabolic disorders. The prediction ability for healthy newborns was high (81.82% of correctly classified cases), and two samples were found as belonging to either the healthy or disorder group. Finally, the prediction ability was excellent for newborns suffering from metabolic disorders (90.91%) and only one sample was not identified as belonging to either healthy newborns or newborns with diseases class, and it was recognized as an independent sample.

**Table 6.** Classification of DBS samples results into two groups (healthy newborns and newborns suffering from metabolic disorders) after SIMCA

		Predicted group membership		
Actual group	No. of cases	% Classification	Group H (healthy)	Group D (metabolic disorders)
Training set				
Group H (healthy)	31	83.87	30	5^a^
Group D (metabolic disorders)	31	80.65	31	5^b^
Prediction set				
Group H (healthy)	11	81.82	11	2^c^
Group D (metabolic disorders)	11	90.91	10^d^	0

^a^Four cases as belonging to Group H and Group D, and one misclassified case.

^b^Five cases as belonging to Group H and Group D.

^c^Two cases as belonging to Group H and Group D.

^d^One case identified as a new class.

## Conclusions

Multi-element determination in DBSs from healthy newborns and newborns suffering from metabolic disorders by LA˗ICP-MS shows the following key conclusions:

- LA-ICP-MS analysis of DBSs from available metabolic disorder screening programs can be easily performed to assess trace elements at few µg (mg) l^−1^ in newborns’ blood (DBSs from newborns).
- The use of trace element data allows to distinguish healthy newborns and newborns suffering from metabolic disorders using chemometric tools such as PCA, CA, LDA, and SIMCA. In addition, element data also allow to distinguish among healthy newborns and newborns suffering from PKU, MSUD, and other metabolic disorders.
- Relationships between several essential elements (K, Fe, Rb, and Zn) and PKU and MSUD biomarkers have been found, which represent a promising avenue to detect metabolic disorders by assessing trace elements in DBSs. In addition, trace element data can give complementary information compared with those offered by current biomarkers.
- Relationships between certain trace elements and some metabolic disorders could also be the basis of further investigations regarding the influence of these trace elements on the disorder's mechanisms.
- Finally, the poor discrimination offered by trace elements when classifying other metabolic disorders can be attributed to the small number of DBS samples available from newborns suffering from these disorders. Only one DBS was analyzed for disorders such as CIT, HCY, HMG, 3-MCCD, LCHAD, MUT, NKH, Tyr, and VLCAD, whereas four DBSs for MCAD were included in the study. Collaborative studies among laboratories coping with NBS programs are therefore needed for verifying the influence of trace elements in rare metabolic disorders.

## Supplementary Material

mfab018_Supplemental_File

## Data Availability

The data underlying this article are available in the article and in its online supplementary material.
