# Supplementary material for: Trace elements in dried blood spots as potential discriminating features for metabolic disorder diagnosis in newborns
Source: Metallomics. 2021 Apr 30;13(5):mfab018. doi: 10.1093/mtomcs/mfab018 (PMC8755940; doi:10.1093/mtomcs/mfab018)
Supplement: mfab018_Supplemental_File [file mfab018_supplemental_file.docx]

**Electronic Supplementary Information (ESI)**

Trace elements in dried blood spots as potential discriminating features for metabolic disorders diagnosis in new-borns

Jorge Moreda–Piñeiro^1^, José A. Cocho^2^, María Luz Couce^2^, Antonio Moreda-Piñeiro^3^, Pilar Bermejo-Barrera^3^

(1) Universidade da Coruña. Department of Chemistry. Faculty of Sciences. Grupo Química Analítica Aplicada (QANAP), University Institute of Research in Environmental Studies (IUMA), Centro de Investigaciones Científicas Avanzadas (CICA), Campus de A Coruña, s/n. 15071 – A Coruña. Spain.

(2) Unit of Diagnosis and Treatment of Congenital Metabolic Diseases, Department of Pediatrics, University Hospital of Santiago de Compostela, IDIS, CIBERER, A Choupana, s/n, 15706 Santiago de Compostela, Spain.

(3) Group of Trace Elements, Speciation and Spectroscopy (GETEE), Strategic Grouping in Materials (AEMAT), Department of Analytical Chemistry, Nutrition and Bromatology. Faculty of Chemistry. Universidade de Santiago de Compostela. Avenida das Ciencias, s/n. 15782, Santiago de Compostela. Spain.

Table S1 Experimental parameters used for LA−ICP-MS

| Operating LA conditions | | |
| --- | --- | --- |
| Laser fluence / J cm^−2^ |  | 3.0 |
| Laser power / % |  | 65 |
| Scan speed / µm s^−1^ |  | 8.0 |
| Repetition rate / Hz |  | 20 |
| Ablation depth / µm |  | 0 |
| Spot diameter / µm |  | 90 |
| Scan pattern |  | Lineal scan |
| Runs |  | 100 |
| Analysis time / s |  | 188 |
| Size of ablated area / mm^2^ |  | 0.135 |
| He flow rate / mL min^−1^ |  | 1.0 |
| Wash up time / s |  | 55 |
| Operating ICP-MS conditions | | |
| Radiofrequency power / W |  | 1475 |
| Gas flows / L min^-1^ | Cooling | 15 |
|  | Auxiliary | 0.90 |
|  | Additional | 0.97 |
| Mass-to- charge ratio | Ca, Cu, Fe, K, Mg, Na, P, Pb, Rb, Sb, Sr and Zn | 44, 63, 56, 39, 24, 23, 31, 208, 85, 121, 88 and 66 |
| Resolution mode | Low |  |

**Table S2** New-born data and amino acids and fatty acid concentrations in several DBSs from new-borns.

|  | **Metabolic disease detected** | **Newborn weight** | **Birth days after sample collection** | **Propionyl** | **Hexanoyl** | **Octanoyl** | **3Methil- Glutaryl** | **C14:1** | **C14-OH** | **C16-OH** | **Glycine** | **Valine** | **Leucine/Alanine**  **Ratio** | **Citruline** | **Phenylalanine** | **Phenylalanine/Tyrosine Ratio** |
| --- | --- | --- | --- | --- | --- | --- | --- | --- | --- | --- | --- | --- | --- | --- | --- | --- |
|  |  | g | Days | µmol L^-1^ | | | | | | | | | | | | |
| **S1** | ˗^a^ | 3690 | 3 | 1.4 | 0.13 | 0.07 | 0.10 | 0.06 | 0.03 | 0.07 | 525.6 | 157.5 | 0.49 | 12.0 | 67.0 | 0.55 |
| **S2** | ˗^a^ | 3640 | 2 | 1.0 | 0.03 | 0.06 | 0.10 | 0.13 | 0.04 | 0.05 | 243.3 | 100.1 | 0.70 | 13.2 | 47.8 | 0.65 |
| **S3** | ˗^a^ | 3445 | 3 | 2.7 | 0.12 | 0.11 | 0.08 | 0.14 | 0.03 | 0.05 | 260.0 | 134.8 | 0.88 | 11.9 | 58.9 | 0.91 |
| **S4** | ˗^a^ | 3940 | 3 | 2.1 | 0.17 | 0.13 | 0.06 | 0.17 | 0.04 | 0.08 | 228.2 | 82.5 | 0.55 | 9.4 | 50.4 | 1.1 |
| **S5** | ˗^a^ | 3135 | 3 | 1.4 | 0.13 | 0.10 | 0.08 | 0.04 | 0.06 | 0.07 | 284.3 | 114.4 | 0.59 | 11.2 | 52.3 | 0.78 |
| **S6** | ˗^a^ | 3950 | 7 | 2.4 | 0.16 | 0.14 | 0.15 | 0.18 | 0.05 | 0.06 | 326.8 | 116.3 | 0.69 | 9.8 | 60.4 | 0.98 |
| **S7** | ˗^a^ | 3450 | 5 | 1.2 | 0.13 | 0.11 | 0.07 | 0.17 | 0.05 | 0.07 | 526.9 | 188.7 | 0.74 | 15.0 | 86.4 | 0.83 |
| **S8** | ˗^a^ | 2720 | 15 | 1.5 | 0.13 | 0.14 | 0.07 | 0.08 | 0.08 | 0.04 | 245.3 | 106.0 | 0.52 | 9.3 | 43.2 | 0.71 |
| **S9** | ˗^a^ | 3550 | 3 | 2.2 | 0.12 | 0.13 | 0.06 | 0.11 | 0.11 | 0.05 | 410.3 | 158.7 | 0.58 | 18.7 | 68.6 | 0.42 |
| **S10** | ˗^a^ | 3010 | 3 | 2.5 | 0.12 | 0.11 | 0.13 | 0.11 | 0.08 | 0.06 | 361.2 | 146.5 | 0.51 | 9.4 | 52.3 | 0.51 |
| **S11** | ˗^a^ | 3550 | 3 | 2.7 | 0.12 | 0.14 | 0.08 | 0.14 | 0.03 | 0.03 | 356.2 | 138.5 | 0.59 | 10.9 | 57.3 | 0.55 |
| **S12** | ˗^a^ | 2890 | 3 | 2.7 | 0.18 | 0.14 | 0.07 | 0.09 | 0.04 | 0.06 | 344.7 | 131.1 | 0.73 | 14.8 | 82.9 | 0.57 |
| **S13** | ˗^a^ | 3970 | 14 | 1.8 | 0.12 | 0.14 | 0.17 | 0.27 | 0.06 | 0.06 | 225.9 | 112.7 | 0.76 | 8.2 | 46.8 | 0.82 |
| **S14** | ˗^a^ | 3360 | 3 | 2.0 | 0.16 | 0.04 | 0.18 | 0.17 | 0.04 | 0.11 | 247.9 | 128.8 | 0.66 | 11.3 | 42.8 | 0.47 |
| **S15** | ˗^a^ | 3560 | 3 | 1.5 | 0.10 | 0.12 | 0.14 | 0.17 | 0.02 | 0.09 | 248.1 | 101.7 | 0.58 | 10.7 | 40.4 | 0.59 |
| **S16** | ˗^a^ | 2610 | 3 | 1.6 | 0.15 | 0.12 | 0.10 | 0.15 | 0.07 | 0.05 | 223.6 | 62.5 | 0.50 | 8.5 | 35.9 | 0.81 |
| **S17** | ˗^a^ | 3500 | 3 | 2.7 | 0.16 | 0.11 | 0.11 | 0.14 | 0.09 | 0.06 | 383.9 | 128.0 | 0.55 | 14.8 | 57.5 | 0.73 |
| **S18** | ˗^a^ | 2770 | 2 | 1.7 | 0.15 | 0.13 | 0.09 | 0.12 | 0.09 | 0.07 | 446.3 | 206.4 | 0.59 | 14.7 | 91.8 | 0.77 |
| **S19** | ˗^a^ | 3920 | ˗^b^ | 1.2 | 0.15 | 0.08 | 0.08 | 0.09 | 0.03 | 0.05 | 318.5 | 137.3 | 0.73 | 9.9 | 64.0 | 0.97 |
| **S20** | ˗^a^ | 3215 | 4 | 4.7 | 0.19 | 0.08 | 0.13 | 0.24 | 0.06 | 0.04 | 224.1 | 104.9 | 0.66 | 8.5 | 53.9 | 1.0 |
| **S21** | ˗^a^ | 3275 | 10 | 1.3 | 0.21 | 0.12 | 0.10 | 0.12 | 0.05 | 0.06 | 258.2 | 137.0 | 0.81 | 14.1 | 73.5 | 1.0 |
| **S22** | ˗^a^ | 3000 | 2 | 1.3 | 0.11 | 0.17 | 0.09 | 0.16 | 0.06 | 0.05 | 269.0 | 117.4 | 0.74 | 9.6 | 46.5 | 0.47 |
| **S23** | ˗^a^ | 1550 | 0 | 1.3 | 0.12 | 0.11 | 0.09 | 0.06 | 0.03 | 0.04 | 247.1 | 81.0 | 0.61 | 12.4 | 43.2 | 0.90 |
| **S24** | ˗^a^ | 3200 | 1 | 2.1 | 0.20 | 0.10 | 0.12 | 0.18 | 0.08 | 0.04 | 366.8 | 111.8 | 0.59 | 16.6 | 51.5 | 0.55 |
| **S25** | ˗^a^ | 3040 | 1 | 1.9 | 0.20 | 0.18 | 0.10 | 0.14 | 0.07 | 0.03 | 280.1 | 117.9 | 0.66 | 9.6 | 53.5 | 0.65 |
| **S26** | ˗^a^ | 3260 | 3 | 1.7 | 0.14 | 0.16 | 0.11 | 0.11 | 0.05 | 0.06 | 316.0 | 141.0 | 0.57 | 11.3 | 60.4 | 0.48 |
| **S27** | ˗^a^ | 3555 | 3 | 1.7 | 0.12 | 0.05 | 0.06 | 0.06 | 0.02 | 0.06 | 247.0 | 153.5 | 0.67 | 11.2 | 45.9 | 0.47 |
| **S28** | ˗^a^ | 3660 | 3 | 1.5 | 0.13 | 0.15 | 0.11 | 0.08 | 0.13 | 0.06 | 264.5 | 159.2 | 0.72 | 12.7 | 49.3 | 0.56 |
| **S29** | ˗^a^ | 2780 | 8 | 2.8 | 0.12 | 0.09 | 0.10 | 0.14 | 0.08 | 0.06 | 251.6 | 119.9 | 0.67 | 14.1 | 56.1 | 0.57 |
| **S30** | ˗^a^ | 3100 | ˗^b^ | 2.2 | 0.10 | 0.13 | 0.04 | 0.18 | 0.06 | 0.07 | 557.2 | 120.4 | 0.53 | 12.0 | 71.8 | 0.94 |
| **S31** | ˗^a^ | 3275 | 2 | 0.68 | 0.19 | 0.18 | 0.09 | 0.05 | 0.03 | 0.06 | 188.5 | 86.3 | 0.72 | 7.8 | 43.1 | 0.72 |
| **S32** | ˗^a^ | 3705 | 3 | 2.2 | 0.10 | 0.10 | 0.10 | 0.11 | 0.04 | 0.05 | 216.5 | 118.1 | 0.72 | 10.8 | 48.9 | 1.1 |
| **S33** | ˗^a^ | 3030 | 2 | 1.8 | 0.14 | 0.12 | 0.10 | 0.13 | 0.05 | 0.05 | 257.0 | 116.8 | 0.83 | 13.4 | 55.7 | 0.72 |
| **S34** | ˗^a^ | 3320 | 3 | 1.7 | 0.11 | 0.10 | 0.09 | 0.10 | 0.07 | 0.05 | 232.5 | 84.3 | 0.73 | 9.7 | 53.4 | 0.87 |
| **S35** | ˗^a^ | 3655 | 15 | 1.2 | 0.11 | 0.14 | 0.09 | 0.09 | 0.01 | 0.04 | 184.0 | 156.3 | 0.78 | 10.2 | 48.5 | 0.24 |
| **S36** | ˗^a^ | 3605 | 5 | 2.1 | 0.11 | 0.12 | 0.13 | 0.19 | 0.10 | 0.04 | 329.1 | 129.7 | 0.53 | 13.8 | 54.6 | 0.70 |
| **S37** | ˗^a^ | 2435 | 6 | 0.52 | 0.09 | 0.12 | 0.08 | 0.07 | 0.04 | 0.06 | 269.9 | 141.5 | 0.53 | 28.9 | 72.6 | 0.72 |
| **S38** | ˗^a^ | 4110 | 5 | 1.7 | 0.20 | 0.09 | 0.08 | 0.15 | 0.09 | 0.09 | 328.7 | 126.9 | 0.67 | 7.6 | 50.6 | 0.57 |
| **S39** | ˗^a^ | 3760 | 5 | 2.2 | 0.12 | 0.24 | 0.27 | 0.39 | 0.08 | 0.10 | 270.7 | 120.6 | 0.68 | 13.4 | 52.4 | 0.49 |
| **S40** | ˗^a^ | 3090 | 4 | 3.5 | 0.17 | 0.12 | 0.10 | 0.18 | 0.03 | 0.04 | 311.8 | 165.0 | 0.87 | 11.3 | 90.7 | 0.76 |
| **S41** | MCAD | 1950 | 3 | 2.0 | 0.09 | 0.07 | 0.03 | 0.03 | 0.07 | 0.06 | 139.2 | 164.9 | 0.41 | 11.3 | 51.4 | 0.75 |
| **S42** | ˗^a^ | 3450 | 0 | 2.3 | 0.14 | 0.11 | 0.05 | 0.05 | 0.04 | 0.08 | 227.4 | 211.8 | 0.83 | 24.2 | 59.5 | 0.80 |
| **S43** | ˗^a^ | 3520 | ˗^b^ | 1.0 | 0.06 | 0.09 | 0.03 | 0.09 | 0.03 | 0.01 | 160.4 | 111.5 | 0.68 | 17.9 | 315.9 | 11.4 |
| **S44** | MSUD | 2810 | 1 | 0.91 | 0.14 | 0.17 | 0.06 | 0.08 | 0.02 | 0.02 | 398.2 | 105.1 | 0.51 | 15.9 | 82.0 | 0.86 |
| **S45** | PKU | 3560 | ˗^b^ | 2.0 | 0.11 | 0.06 | 0.05 | 0.03 | 0.01 | 0.06 | 153.5 | 137.6 | 0.54 | 19.1 | 263.9 | 7.0 |
| **S46** | MUT | 2770 | 0 | 1.1 | 0.12 | 0.06 | 0.04 | 0.02 | 0.03 | 0.05 | 144.1 | 98.1 | 0.52 | 24.8 | 57.1 | 1.1 |
| **S47** | MSUD | 3420 | 1 | 1.9 | 0.07 | 0.22 | 0.05 | 0.08 | 0.04 | 0.02 | 165.0 | 125.0 | 0.56 | 10.9 | 138.4 | 2.8 |
| **S48** | Citr I | 3075 | 0 | 2.3 | 0.20 | 0.17 | 0.10 | 0.09 | 0.02 | 0.03 | 138.6 | 175.6 | 0.50 | 181.7 | 76.9 | 0.84 |
| **S49** | VLCAD | 3325 | ˗^b^ | 0.92 | 0.11 | 0.02 | 0.09 | 2.06 | 0.06 | 0.09 | 154.1 | 130.9 | 0.60 | 17.5 | 57.6 | 0.90 |
| **S50** | PKU | 3540 | 1 | 0.77 | 0.09 | 0.06 | 0.06 | 0.22 | 0.11 | 0.03 | 124.1 | 162.2 | 1.5 | 8.0 | 496.6 | 17.3 |
| **S51** | MSUD | 2810 | 1 | 2.3 | 0.13 | 0.20 | 0.04 | 0.07 | 0.01 | <LOQ | 292.5 | 159.5 | 0.53 | 22.7 | 71.3 | 0.88 |
| **S52** | MSUD | 3800 | 2 | 1.5 | 0.12 | 0.15 | 0.04 | 0.05 | 0.04 | 0.03 | 237.4 | 101.5 | 0.41 | 16.4 | 63.6 | 0.61 |
| **S53** | MSUD | ˗^b^ | 1 | 1.7 | 0.07 | 0.05 | 0.02 | 0.06 | 0.03 | 0.04 | 145.0 | 171.7 | 1.7 | 14.8 | 32.5 | 1.1 |
| **S54** | PKU | ˗^b^ | ˗^b^ | 1.5 | 0.02 | 0.13 | 0.06 | 0.11 | 0.03 | 0.04 | 185.9 | 130.5 | 0.56 | 22.0 | 736.0 | 18.3 |
| **S55** | PKU | 3190 | 1 | 1.5 | 0.11 | 0.18 | 0.07 | 0.12 | 0.05 | 0.07 | 169.3 | 120.5 | 0.52 | 11.2 | 84.6 | 3.5 |
| **S56** | PKU | 3540 | 1 | 1.5 | 0.03 | 0.12 | 0.03 | 0.15 | 0.04 | 0.04 | 206.1 | 146.9 | 0.59 | 24.3 | 255.9 | 6.7 |
| **S57** | PKU | 3000 | 0 | 1.7 | 0.09 | 0.26 | 0.03 | 0.14 | 0.03 | <LOQ | 148.5 | 124.8 | 0.74 | 21.6 | 253.0 | 5.6 |
| **S58** | PKU | 3620 | ˗^b^ | 2.3 | 0.12 | 0.17 | 0.06 | 0.12 | 0.04 | <LOQ | 117.8 | 165.1 | 0.40 | 20.6 | 215.4 | 5.7 |
| **S59** | PKU | 3620 | ˗^b^ | 2.0 | 0.10 | 0.03 | 0.07 | 0.04 | <LOQ | 0.01 | 91.3 | 135.0 | 0.44 | 14.4 | 166.0 | 5.0 |
| **S60** | MSUD | ˗^b^ | 1 | 1.7 | 0.12 | 0.11 | 0.02 | 0.05 | 0.03 | 0.06 | 258.1 | 282.9 | 1.8 | 20.8 | 55.2 | 0.84 |
| **S61** | PKU | ˗^b^ | ˗^b^ | 1.1 | 0.04 | 0.03 | 0.06 | 0.06 | 0.01 | 0.01 | 178.0 | 134.1 | 0.42 | 21.0 | 264.1 | 6.3 |
| **S62** | MSUD | ˗^b^ | 2 | 2.1 | 0.13 | 0.16 | 0.04 | 0.14 | 0.08 | 0.09 | 303.6 | 305.5 | 2.90 | 29.1 | 69.0 | 0.65 |
| **S63** | PKU | 3460 | ˗^b^ | 2.1 | 0.14 | 0.10 | 0.05 | 0.09 | 0.09 | 0.06 | 177.1 | 191.5 | 0.60 | 19.9 | 392.0 | 7.6 |
| **S64** | MCAD | 3760 | 0 | 1.3 | 0.73 | 1.6 | 0.05 | 0.08 | 0.03 | 0.09 | 164.3 | 117.8 | 0.69 | 28.4 | 65.5 | 1.1 |
| **S65** | MSUD | 3420 | 1 | 1.9 | 0.14 | 0.13 | 0.05 | 0.08 | 0.04 | 0.06 | 341.8 | 135.4 | 0.26 | 17.2 | 123.9 | 0.67 |
| **S66** | MSUD | 2810 | ˗^b^ | 2.2 | 0.17 | 0.10 | 0.11 | 0.12 | 0.02 | 0.05 | 290.8 | 201.3 | 0.44 | 23.4 | 74.7 | 0.83 |
| **S67** | MSUD | 3730 | 1 | 1.3 | 0.16 | 0.06 | 0.05 | 0.06 | <LOQ | 0.03 | 194.6 | 209.5 | 1.85 | 18.0 | 43.6 | 0.68 |
| **S68** | PKU | 3600 | 2 | 1.5 | 0.08 | 0.08 | 0.05 | 0.08 | 0.05 | 0.02 | 217.5 | 164.8 | 0.50 | 20.6 | 482.2 | 9.0 |
| **S69** | LCHAD | 3240 | ˗^b^ | 0.93 | 0.06 | 0.07 | 0.18 | 0.92 | 0.37 | 0.95 | 150.0 | 120.1 | 0.58 | 24.2 | 65.6 | 0.85 |
| **S70** | PKU | ˗^b^ | 0 | 2.5 | 0.15 | 0.08 | 0.05 | 0.16 | 0.07 | 0.06 | 131.6 | 138.7 | 0.50 | 16.6 | 382.0 | 10.6 |
| **S71** | HMG | 3470 | 0 | 2.9 | 0.21 | 0.06 | 0.84 | 0.10 | 0.05 | 0.04 | 363.3 | 298.4 | 0.23 | 21.3 | 89.7 | 0.60 |
| **S72** | PKU | 1900 | 0 | 2.4 | 0.11 | 0.24 | 0.06 | 0.20 | 0.09 | 0.03 | 177.3 | 167.9 | 0.49 | 34.5 | 1427 | 35.6 |
| **S73** | MSUD | 3800 | 0 | 1.9 | 0.21 | 0.16 | 0.10 | 0.13 | 0.02 | 0.03 | 254.7 | 109.1 | 0.40 | 32.8 | 118.3 | 1.17 |
| **S74** | PKU | 4070 | 0 | 2.4 | 0.13 | 0.08 | 0.01 | 0.18 | 0.05 | 0.04 | 105.1 | 157.2 | 0.85 | 22.2 | 418.5 | 11.3 |
| **S75** | NKH | 3450 | 0 | 2.0 | 0.11 | 0.07 | 0.05 | 0.13 | 0.03 | 0.03 | 296.6 | 142.1 | 0.40 | 58.8 | 77.0 | 1.3 |
| **S76** | PKU | ˗^b^ | 1 | 2.1 | 0.04 | 0.04 | 0.10 | 0.06 | 0.05 | 0.06 | 240.5 | 152.0 | 0.49 | 35.9 | 498.2 | 7.1 |
| **S77** | PKU | 3190 | 1 | 1.6 | 0.15 | 0.24 | 0.05 | 0.14 | 0.04 | 0.06 | 270.2 | 158.1 | 0.45 | 15.8 | 28.4 | 0.37 |
| **S78** | PKU | 1630 | 0 | 1.0 | 0.07 | 0.07 | 0.05 | 0.06 | 0.01 | 0.04 | 250.7 | 119.6 | 0.35 | 26.2 | 376.4 | 8.1 |
| **S79** | MCAD | 2750 | 3 | 0.70 | 0.65 | 3.4 | 0.09 | 0.07 | 0.09 | 0.05 | 457.5 | 230.2 | 0.53 | 48.2 | 85.8 | 0.85 |
| **S80** | TYR | ˗^b^ | 1 | 1.7 | 0.14 | 0.06 | 0.20 | 0.32 | 0.06 | 0.05 | 226.8 | 181.5 | 0.38 | 35.0 | 532.7 | 10.5 |
| **S81** | MCAD | 2730 | ˗^b^ | 2.7 | 1.4 | 2.35 | 0.09 | 0.08 | 0.07 | 0.07 | 192.7 | 177.8 | 0.56 | 18.0 | 80.9 | 1.1 |
| **S82** | PKU | ˗^b^ | ˗^b^ | 1.7 | 0.10 | 0.13 | 0.06 | 0.03 | 0.02 | 0.03 | 278.8 | 175.8 | 0.43 | 48.2 | 1009 | 20.6 |
| **S83** | HCY | 3340 | 0 | 2.7 | 0.10 | 0.19 | 0.02 | 0.09 | 0.03 | 0.03 | 148.5 | 190.0 | 0.65 | 27.2 | 47.5 | 0.78 |
| **S84** | MCCD | 3470 | 0 | 2.2 | 0.14 | 0.08 | 0.67 | 0.04 | 0.03 | 0.03 | 377.0 | 257.4 | 0.26 | 24.9 | 77.8 | 0.78 |
| ˗^a^ Healthy  ˗^b^ Data not available  C14:1: Tetradecenoylcarnitine; C14-OH: 3-Hydroxytetradecanoylcarnitine, C16-OH: 3-Hydroxypalmitoylcarnitine  CIT: Citrullinemia Type I; HCY: Homocystinuria; HMG: Hydroxymethyl glutaric aciduria; MCAD: Medium-chain acyl-CoA dehydrogenase deficiency; 3-MCCD: 3-methylcrotonyl-CoA carboxylase deficiency; LCHAD: Long chain 3-hydroxyacyl-CoA dehydrogenase deficiency; MUT: Methylmalonic academia; MSUD: Maple syrup urine disease; NKH: Non-ketotic hyperglycinemia; PKU: Phenylketonuria; TYR: tyrosinemia; VLCAD: Very long-chain 3-hydroxyacyl-CoA dehydrogenase.  Propionyl, hexanoyl and octanoyl: target analytes linked with MCAD; 3 methyl-glutaril: target analyte linked with HMG; C14:1: target analyte linked with VLCAD; C14-OH and C16-OH: target analytes linked with LCHAD; glycine: target analyte linked with NKH; vealine and leucine/valine ratio: target analytes linked with MSUD; citruline: target analyte linked with CIT; and phenylalanine and phenylalanine/tyrosine ratio: target analytes linked with PKU. | | | | | | | | | | | | | | | | |

**Table S3** Element concentrations in several DBSs from new-borns

|  | **Ca** | **Cu** | **Fe** | **K** | **Mg** | **Na** | **P** | **Pb** | **Rb** | **Sb** | **Sr** | **Zn** |
| --- | --- | --- | --- | --- | --- | --- | --- | --- | --- | --- | --- | --- |
|  | mg L^−1^ | | | | | | | µg L^−1^ | mg L^−1^ | µg L^−1^ | | mg L^−1^ |
| **S1** | <13.1 | 0.81 ± 0.090 | 830.0± 74.7 | 3880 ± 465.5 | <11.9 | 2169 ± 260.2 | 658.9 ± 79.1 | <7.0 | 3.1 ± 0.31 | <1.6 | 17.7 ± 2.1 | 2.1 ± 0.19 |
| **S2** | <13.1 | 0.75 ± 0.068 | 675.3± 54.0 | 3284 ± 394.0 | <11.9 | 2186 ± 262.3 | 660.8 ± 79.3 | <7.0 | 35 ± 0.35 | 25.4± 1.2 | 16.6 ± 1.9 | 1.7 ± 0.13 |
| **S3** | <13.1 | 0.83 ± 0.075 | 733.0 ± 36.7 | 3328 ± 399.3 | <11.9 | 2167 ± 260.0 | 793.2 ± 95.1 | <7.0 | 3.3 ± 0.19 | 43.7 ± 4.3 | 16.5 ± 1.9 | 2.4 ± 0.12 |
| **S4** | <13.1 | 0.86 ± 0.068 | 778.7 ± 15.6 | 3920 ± 470.4 | <11.9 | 3089 ± 370.6 | 683.0 ± 34.1 | <7.0 | 4.6 ± 0.64 | <1.6 | 10.4 ± 1.1 | 3.3 ± 0.26 |
| **S5** | <13.1 | 0.96 ± 0.067 | 701.3 ± 56.1 | 3423± 410.7 | <11.9 | 3108 ± 372.9 | 677.2 ± 74.5 | <7.0 | 3.8 ± 0.378 | <1.6 | 10.5 ± 0.84 | 3.0 ± 0.38 |
| **S6** | <13.1 | 0.90 ± 0.072 | 780.3 ± 31.2 | 3691 ± 442.8 | 134.6± 6.7 | 2558 ± 127.9 | 455.4 ± 45.5 | 61.1 ± 8.5 | 5.1 ± 0.35 | 5.1± 0.65 | <3.6 | 4.9 ± 0.39 |
| **S7** | <13.1 | 0.98 ± 0.049 | 850.8 ± 110.6 | 3655 ± 438.6 | 66.3 ± 5.3 | 2507 ± 275.7 | 392.6 ± 23.5 | <7.0 | 5.0 ± 0.55 | <1.6 | <3.6 | 3.0± 0.21 |
| **S8** | <13.1 | 0.90 ± 0.018 | 887.3 ± 97.6 | 3928 ± 471.3 | 22.6 ± 0.90 | 2560 ± 307.2 | 417.5 ± 29.2 | 16.2 ± 2.4 | 3.6 ± 0.25 | <1.6 | 19.8 ± 0.99 | 4.8 ± 0.24 |
| **S9** | <13.1 | 0.97 ± 0.048 | 1044 ± 83.5 | 4558± 546.9 | 28.6 ± 3.1 | 2105 ± 168.4 | 499.0 ± 39.9 | 14.0 ± 1.3 | 5.2 ± 0.36 | 2.0± 0.22 | 22.2 ± 2.6 | 3.6 ± 0.39 |
| **S10** | <13.1 | 0.91 ± 0.091 | 737.1 ± 58.9 | 3220 ± 386.4 | 79.6 ± 5.5 | 1980 ± 198.0 | 433.2 ± 43.3 | 25.3± 2.7 | 3.5 ± 0.35 | 12.9 ± 1.5 | 8.5 ± 0.85 | 2.6 ± 0.30 |
| **S11** | <13.1 | 0.85 ± 0.093 | 637.1 ± 44.5 | 2775 ± 332.9 | 98.9 ± 4.9 | 2628 ± 210.2 | 429.7 ± 30.0 | 10.7± 1.1 | 3.4 ± 0.44 | <1.6 | 14.2 ± 1.7 | 3.1± 0.18 |
| **S12** | <13.1 | 0.82 ± 0.066 | 737.9 ± 44.3 | 3186± 382.2 | 62.4 ± 6.8 | 1838 ± 110.3 | 498.0 ± 19.9 | 19.1 ± 1.7 | 3.5 ± 0.21 | 6.3 ± 0.63 | 15.6 ± 0.93 | 2.3 ± 0.23 |
| **S13** | <13.1 | 0.81 ± 0.040 | 519.6 ± 25.9 | 2426± 291.1 | 44.0 ± 5.7 | 2416 ± 193.2 | 371.8 ± 33.4 | 14.5 ± 1.1 | 2.0 ± 0.16 | <1.6 | 17.1 ± 1.5 | 1.6 ± 0.049 |
| **S14** | <13.1 | 0.74 ± 0.037 | 709.3 ± 78.0 | 2796 ± 335.4 | 83.1 ± 7.4 | 1628 ± 113.9 | 415.9 ± 54.0 | 15.9 ± 0.95 | 2.4 ± 0.19 | 2.3 ± 0.20 | 12.9 ± 1.5 | 2.2 ± 0.10 |
| **S15** | <13.1 | 0.75 ± 0.030 | 697.0 ± 76.6 | 2848 ± 256.3 | 81.0 ± 3.2 | 1944 ± 213.8 | 455.62 ± 31.8 | 8.2± 1.1 | 3.3 ± 0.23 | <1.6 | 20.5 ± 2.0 | 2.2 ± 0.22 |
| **S16** | <13.1 | 0.99 ± 0.099 | 846.7 ± 101.6 | 3729 ± 335.6 | 86.3 ± 4.3 | 2548 ± 203.8 | 463.3 ± 50.9 | 9.3 ± 1.3 | 2.9 ± 0.17 | <1.6 | 9.3 ± 1.2 | 3.9 ± 0.31 |
| **S17** | <13.1 | 1.3 ± 0.16 | 729.3 ± 94.8 | 3408± 170.38 | 58.7 ± 4.67 | 2802 ± 196.1 | 576.4 ± 46.1 | <7.0 | 3.0 ± 0.29 | 5.1 ± 0.51 | 20.9 ± 2.3 | 3.0 ± 0.12 |
| **S18** | <13.1 | 1.1 ± 0.011 | 806.8 ± 48.4 | 3849 ± 423.3 | 41.97 ± 4.17 | 2403 ± 120.1 | 662.3 ± 39.7 | <7.0 | 3.4 ± 0.27 | 15.3 ± 0.91 | <3.6 | 3.7 ± 0.26 |
| **S19** | <13.1 | 0.89 ± 0.12 | 790.9 ± 71.2 | 3743 ± 374.3 | 43.6 ± 3.4 | 2010 ± 201.0 | 722.9 ± 50.6 | <7.0 | 4.0 ± 0.20 | 39.4 ± 2.8 | 8.1 ± 0.89 | 3.0 ± 0.31 |
| **S20** | <13.1 | 0.81 ± 0.090 | 830.0 ± 83.0 | 3880± 426.7 | <11.9 | 2169 ± 21.6 | 658.9 ± 39.5 | <7.0 | 3.1 ± 0.16 | <1.6 | 17.7 ± 1.9 | 2.1 ± 0.15 |
| **S21** | <13.1 | 1.1 ± 0.15 | 1207 ± 48.3 | 5711± 399.7 | <11.9 | 1373 ± 96.0 | 547.9 ± 38.4 | 42.4± 4.2 | 4.5 ± 0.36 | 2.9 ± 0.28 | <3.6 | 2.2 ± 0.16 |
| **S22** | <13.1 | 0.75 ± 0.082 | 675.3 ± 20.2 | 3284 ± 361.2 | <11.9 | 2186± 109.3 | 660.8 ± 26.4 | <7.0 | 3.5 ± 0.24 | 25.4± 2.7 | 16.6 ± 1.5 | 1.8 ± 0.085 |
| **S23** | <13.1 | 0.87 ± 0.087 | 863.0± 69.0 | 3957 ± 474.8 | 125.6 ± 12.5 | 2383 ± 95.3 | 471.0 ± 33.0 | 15.4 ± 2.6 | 3.9 ± 0.078 | <1.6 | <3.6 | 2.6 ± 0.16 |
| **S24** | <13.1 | 0.83 ± 0.066 | 869.2 ± 43.4 | 3722 ± 483.9 | 102.5 ± 8.2 | 1538 ± 123.0 | 506.8 ± 65.9 | <7.0 | 3.7 ± 0.22 | 9.4 ± 1.1 | 6.2 ± 0.68 | 7.1 ± 0.50 |
| **S25** | 15.4 ± 1.8 | 0.81 ± 0.065 | 896.9 ± 89.6 | 3503 ± 280.2 | 230.1± 13.8 | 1558 ± 140.2 | 483.1 ± 33.8 | 15.8 ± 1.1 | 4.0 ± 0.20 | 12.9 ± 1.5 | 6.2 ± 0.62 | 3.6 ± 0.22 |
| **S26** | <13.1 | 1.3 ± 0.089 | 1416 ± 84.9 | 5351 ± 535.1 | 152.2± 10.6 | 2516 ± 226.3 | 556.6 ± 28.3 | 22.1 ± 0.22 | 4.5 ± 0.32 | 4.8 ± 0.28 | 12.8 ± 1.4 | 4.4 ± 0.48 |
| **S27** | <13.1 | 1.1 ± 0.10 | 1115 ± 89.2 | 4908 ± 392.63 | 91.7 ± 6.4 | 2565 ± 205.1 | 530.1 ± 26.5 | 226.9 ± 24.9 | 6.3 ± 0.75 | 4.1 ± 0.28 | 6.3 ± 0.38 | 4.1 ± 0.49 |
| **S28** | <13.1 | 1.2 ± 0.083 | 1218 ± 73.0 | 5254 ± 525.3 | 89.4 ± 4.4 | 2765 ± 55.3 | 575.1 ± 5.8 | 14.2 ± 1.13 | 5.4 ± 0.64 | 7.5 ± 0.67 | 7.2 ± 0.58 | 6.6 ± 0.86 |
| **S29** | <13.1 | 0.75 ± 0.083 | 762.6 ± 30.5 | 3418 ± 205.0 | 77.6 ± 4.6 | 2600 ± 337.9 | 246.4 ± 43.7 | <7.0 | 3.7 ± 0.45 | 3.9± 0.31 | 17.4 ± 1.7 | 3.2 ± 0.16 |
| **S30** | <13.1 | <0.60 | 879.6 ± 61.5 | 3769 ± 301.4 | <11.9 | 1411 ± 112.8 | 717.8 ± 79.0 | <7.0 | 3.3 ± 0.26 | 44.8 ± 5.3 | 15.3 ± 1.8 | 2.5 ± 0.25 |
| **S31** | <13.1 | 1.0 ± 0.061 | 977.7 ± 97.7 | 4121 ± 247.2 | 31.2 ± 3.7 | 2990 ± 209.3 | 693.3 ± 6.9 | <7.0 | 3.9 ± 0.24 | <1.6 | <3.6 | 4.8 ± 0.53 |
| **S32** | <13.1 | 1.0 ± 0.072 | 820.5 ± 65.6 | 3623 ± 362.2 | 16.5 ± 1.9 | 3210± 160.5 | 552.5 ± 44.2 | <7.0 | 3.7 ± 0.41 | <1.6 | <3.6 | 2.7 ± 0.27 |
| **S33** | <13.1 | 0.84 ± 0.092 | 779.2 ± 54.5 | 3890 ± 311.2 | 40.7 ± 4.8 | 2048 ± 225.3 | 598.3 ± 47.9 | 17.9 ± 1.2 | 4.5 ± 0.23 | 15.5± 1.8 | 19.2 ± 2.3 | 2.0 ± 0.20 |
| **S34** | <13.1 | 0.78 ± 0.078 | 725.8 ± 58.1 | 3355 ± 268.4 | 41.9 ± 5.0 | 2475 ± 148.5 | 554.5 ± 38.8 | 47.6 ± 2.8 | 4.2 ± 0.29 | 8.4± 0.91 | 19.8 ± 1.8 | 2.0 ± 0.079 |
| **S35** | <13.1 | 1.1 ± 0.12 | 869.8 ± 60.9 | 2901 ± 232.1 | 80.1 ± 9.6 | 2698 ± 242.9 | 436.4 ± 21.8 | 19.0 ± 1.3 | 2.8 ± 0.11 | 1.6 ± 0.13 | 5.8 ± 0.64 | 10.9 ± 0.88 |
| **S36** | <13.1 | 0.97 ± 0.11 | 868.5 ± 78.1 | 4232 ± 253.8 | 68.9 ± 8.2 | 2823 ± 225.83 | 467.3 ± 28.0 | <7.0 | 5.1 ± 0.45 | 4.6 ± 0.45 | 8.8 ± 0.70 | 3.0± 0.21 |
| **S37** | 15.0 ± 1.4 | 1.0± 0.12 | 587.1 ± 29.3 | 1585 ± 158.4 | 82.3 ± 9.8 | 2808 ± 280.73 | 411.7 ± 28.8 | 34.8 ± 3.8 | 0.89 ± 0.071 | 10.0 ± 0.90 | 15.1 ± 1.6 | 2.8 ± 0.28 |
| **S38** | <13.1 | 0.83 ± 0.091 | 891.6 ± 80.2 | 3766 ± 338.9 | 218.3 ± 26.1 | 22513± 135.03 | 509.0 ± 35.6 | <7.0 | 3.2 ± 0.34 | 4.7 ± 0.41 | 19.8 ± 1.9 | 3.2 ± 0.26 |
| **S39** | <13.1 | 1.1 ± 0.15 | 951.2 ± 57.0 | 3851 ± 231.0 | 229.6 ± 20.6 | 2899 ± 173.93 | 482.5 ± 38.5 | <7.0 | 4.2 ± 0.33 | 3.3 ± 0.19 | 14.2 ± 1.7 | 3.6 ± 0.071 |
| **S40** | <13.1 | 1.3 ± 0.067 | 1122 ± 112.2 | 4158 ± 415.7 | 419.0 ± 46.0 | 32683 ± 326.83 | 548.6 ± 49.3 | 11.0± 0.77 | 4.9 ± 0.29 | 5.0 ± 0.049 | <3.6 | 3.4 ± 0.45 |
| **S41** | <13.1 | 1.6 ± 0.078 | 616.8 ± 43.1 | 2926 ± 234.1 | 243.8 ± 24.3 | 36153 ± 253.03 | 396.2 ± 19.8 | 15.9 ± 0.95 | 4.6 ± 0.31 | <1.6 | 9.4 ± 0.56 | 5.5 ± 0.44 |
| **S42** | <13.1 | 1.3 ± 0.051 | 845.1 ± 50.7 | 2960 ± 59.1 | 254.9 ± 28.0 | 31963± 287.63 | 360.2 ± 7.2 | 9.2 ± 0.65 | 1.3 ± 0.17 | <1.6 | 11.2 ± 1.3 | 9.7 ± 0.58 |
| **S43** | <13.1 | 0.76± 0.046 | 603.0 ± 42.2 | 2470 ± 321.0 | 57.5 ± 6.8 | 2485 ± 149.0 | 388.5 ± 15.5 | <7.0 | 3.2 ± 0.31 | <1.6 | 14.5 ± 1.1 | 5.2 ± 0.36 |
| **S44** | <13.1 | 1.4 ± 0.11 | 435.7 ± 47.9 | 2757 ± 220.5 | <11.9 | 1991 ± 119.4 | 325.8 ± 26.1 | 55.1 ± 3.3 | 0.69 ± 0.075 | 13.9± 1.5 | <3.6 | 5.7 ± 0.34 |
| **S45** | <13.1 | 2.0 ± 0.18 | 801.0 ± 96.1 | 3491 ± 244.3 | <11.9 | 1872 ± 243.3 | 363.9 ± 25.4 | 26.0 ± 2.6 | 3.0 ± 0.38 | 6.3 ± 0.37 | <3.6 | 5.4 ± 0.27 |
| **S46** | 23.0 ± 2.5 | 8.5 ± 0.76 | 644.4 ± 83.7 | 3113 ± 186.8 | 39.2 ± 3.1 | 3110 ± 186.6 | 375.2 ± 18.7 | <7.0 | 2.9 ± 0.26 | 12.0 ± 1.5 | <3.6 | 7.3 ± 0.72 |
| **S47** | <13.1 | 1.1 ± 0.11 | 491.3 ± 24.5 | 2652 ± 318.2 | 16.3 ± 1.6 | 2357 ± 164.9 | 260.5 ± 28.6 | 43.1 ± 3.8 | 0.60 ± 0.024 | 6.7 ± 0.73 | <3.6 | 4.1 ± 0.20 |
| **S48** | <13.1 | 1.3 ± 0.13 | 576.8 ± 34.6 | 2974 ± 118.9 | 26.3 ± 2.6 | 2639 ± 263.9 | 266.3 ± 34.6 | 36.3 ± 3.6 | 3.7 ± 0.29 | 3.5 ± 0.35 | <3.6 | 4.3 ± 0.47 |
| **S49** | <13.1 | 1.2± 0.13 | 718.7 ± 71.8 | 3356 ± 268.5 | 24.3 ± 3.1 | 2822 ± 282.2 | 280.8 ± 25.2 | 42.9 ± 5.5 | 3.4 ± 0.37 | 4.7 ± 0.52 | <3.6 | 7.3 ± 0.29 |
| **S50** | 19.4 ± 2.5 | 1.4 0.057 | 478.4 ± 19.1 | 2218 ± 133.0 | 200.5 ± 24.0 | 3006 ± 120.2 | 300.9 ± 12.0 | 64.3 ± 3.8 | 2.1± 0.14 | 6.7 ± 0.60 | <3.6 | 6.5 ± 0.65 |
| **S51** | 19.4 ± 2.1 | 1.1 ± 0.032 | 412.9 ± 28.9 | 1872 ± 149.7 | 23.5 ± 2.1 | 2408 ± 192.6 | 397.7 ± 11.9 | 20.1 ± 2.4 | 0.78 ± 0.10 | 3.6 ± 0.25 | 16.7 ± 1.9 | 5.1 ± 0.031 |
| **S52** | 19.8 ± 2.4 | 1.4 ± 0.10 | 488.1 ± 53.7 | 1954 ± 175.8 | 23.2 ± 2.5 | 2525 ± 277.7 | 469.7 ± 23.4 | 31.9 ± 2.5 | 0.85 ± 0.059 | 2.6 ± 0.33 | 18.2 ± 1.1 | 5.2 ± 0.67 |
| **S53** | 15.6 ± 1.6 | 1.0 ± 0.083 | 491.5 ± 49.1 | 1889 ± 132.2 | 21.2 ± 1.2 | 2352 ± 164.6 | 388.4 ± 23.3 | 40.2 ± 4.0 | 1.5 ± 0.011 | 3.3 ± 0.35 | 13.1 ± 1.2 | 6.1 ± 0.67 |
| **S54** | 15.5 ± 1.6 | 0.74 ± 0.052 | 627.1 ± 62.7 | 2160 ± 259.1 | 25.2 ± 2.3 | 1742 ± 139.3 | 452.6 ± 27.1 | 34.7 ± 2.7 | 2.5 ± 0.20 | 2.2 ± 0.127 | 10.9 ± 0.43 | 5.9 ± 0.46 |
| **S55** | 26.3 ± 2.9 | 2.4 ± 0.12 | 566.1 ± 56.6 | 2071 ± 207.1 | 32.4 ± 3.2 | 2380± 309.3 | 535.7 ± 37.4 | 67.5 ± 4.7 | 0.70 ± 0.035 | 6.6 ± 0.78 | 293.0 ± 26.3 | 5.3 ± 0.57 |
| **S56** | 16.1 ± 1.9 | 1.5 ± 0.16 | 541.5 ± 21.6 | 2234 ± 245.6 | 20.0 ± 0.79 | 2450 ± 196.0 | 505.6 ± 40.4 | 23.1 ± 1.3 | 2.5 ± 0.10 | 3.3 ± 0.42 | 20.7 ± 2.2 | 6.6 ± 0.59 |
| **S57** | 20.3± 2.4 | 1.3 ± 0.13 | 590.3 ± 47.2 | 3166± 253.2 | 29.7 ± 2.3 | 2781 ± 194.6 | 475.1 ± 38.0 | 29.85 ± 1.1 | 2.3 ± 0.16 | <1.6 | 17.4 ± 1.3 | 5.9 ± 0.29 |
| **S58** | 21.4 ± 3.0 | 2.5 ± 0.25 | 736.8 ± 51.5 | 3497 ± 244.8 | 31.5 ± 2.2 | 3244 ± 291.9 | 576.3 ± 28.8 | <7.0 | 3.1 ± 0.34 | 5.6 ± 0.66 | 17.0 ± 1.0 | 9.9 ± 0.98 |
| **S59** | 21.2± 1.7 | 2.2 ± 0.13 | 576.3 ± 63.4 | 2971 ± 237.7 | 27.5 ± 1.6 | 3018 ± 150.9 | 483.9 ± 48.3 | 17.3 ± 1.2 | 2.6 ± 0.18 | <1.6 | 16.7 ± 1.1 | 6.6 ± 0.32 |
| **S60** | 20.7 ± 1.0 | 1.2 ± 0.084 | 466.2 ± 46.6 | 1891 ± 113.4 | 28.0 ± 3.0 | 2326 ± 116.3 | 406.9 ± 44.7 | 28.6 ± 2.8 | 1.2 ± 0.049 | <1.6 | 20.2 ± 1.4 | 3.7 ± 0.43 |
| **S61** | 16.7 ± 1.5 | 0.97 ± 0.078 | 627.6 ± 43.9 | 2332 ± 163.2 | 23.6 ± 0.94 | 2013 ± 221.3 | 454.4 ± 22.7 | 18.7 ± 0.74 | 3.0± 0.29 | <1.6 | 18.1 ± 0.90 | 8.8 ± 1.1 |
| **S62** | 19.7 ± 2.0 | 1.3 ± 0.12 | 731.8 ± 58.5 | 2477 ± 222.9 | 31.0 ± 0.31 | 2416 ± 96.6 | 552.9 ± 22.1 | 67.0 ± 4.6 | 1.7 ± 0.033 | 2.0 ± 0.26 | 17.4 ± 0.17 | 6.2 ± 0.68 |
| **S63** | 22.5 ± 2.5 | 1.2 ± 0.095 | 643.6 ± 12.9 | 2374 ± 118.7 | 28.4 ± 2.2 | 2315 ± 23.1 | 485.0 ± 33.9 | 21.8 ± 1.7 | 2.1 ± 0.16 | <1.6 | 23.3 ± 1.8 | 7.7 ± 0.76 |
| **S64** | 30.1 ± 2.1 | 3.0 ± 0.18 | 517.6 ± 36.2 | 2192 ± 219.2 | 38.7 ± 1.5 | 3553 ± 284.2 | 503.0 ± 50.2 | 159.5 ± 12.7 | 1.3 ± 0.14 | 2.7 ± 0.29 | 25.9 ± 0.25 | 6.7 ± 0.53 |
| **S65** | 17.0 ± 2.0 | 1.4± 0.027 | 533.7 ± 69.4 | 2413 ± 265.4 | 21.3 ± 1.9 | 2543 ± 152.5 | 528.9 ± 21.1 | 23.2 ± 1.15 | 0.87 ± 0.10 | 2.7 ± 0.24 | 12.1 ± 0.96 | 5.0 ± 0.44 |
| **S66** | 22.0 ± 1.8 | 1.2 ± 0.047 | 483.1 ± 38.6 | 2045 ± 81.8 | 25.1 ± 1.8 | 2503 ± 250.2 | 469.7 ± 32.8 | 16.0 ± 0.80 | 0.87 ± 0.086 | <1.6 | 13.2 ± 0.66 | 5.2 ± 0.36 |
| **S67** | 21.0 ± 3.2 | 1.2 ± 0.069 | 570.9 ± 39.9 | 2321 ± 23.2 | 32.7 ± 2.6 | 2654 ± 212.3 | 514.1 ± 56.5 | 19.4 ± 0.19 | <0.60 | 3.9 ± 0.42 | 27.0 ± 1.6 | 13.1 ± 1.0 |
| **S68** | 17.7 ± 1.8 | 1.1 ± 0.079 | 600.9 ± 36.0 | 2067 ± 165.3 | 23.0 ± 1.6 | 2187 ± 153.0 | 400.1 ± 40.0 | 24.8 ± 1.9 | 1.4 ± 0.14 | 2.1 ± 0.16 | 19.0 ± 0.38 | 8.1 ± 0.56 |
| **S69** | 26.4 ± 2.6 | 0.95 ± 0.085 | 529.8 ± 37.1 | 2136 ± 149.5 | 26.5 ± 2.1 | 2393 ± 263.2 | 476.6 ± 33.3 | 17.9 ± 1.1 | 2.6 ± 0.15 | <1.6 | 24.4 ± 1.7 | 6.3 ± 0.12 |
| **S70** | 24.4 ± 2.2 | 1.1 ± 0.10 | 426.2 ± 38.3 | 2389± 167.2 | 29.2 ± 2.0 | 3920 ± 39.2 | 508.5 ± 10.1 | <7.0 | 1.0 ± 0.020 | <1.6 | 18.4 ± 2.0 | 3.8 ± 0.27 |
| **S71** | 19.0 ± 1.1 | 1.5± 0.19 | 598.3 ± 35.8 | 2388 ± 143.3 | 30.1 ± 1.5 | 2544 ± 203.5 | 453.0 ± 31.7 | 8.9 ± 0.97 | 1.3 ± 0.15 | <1.6 | 11.1 ± 0.44 | 4.7 ± 0.33 |
| **S72** | 17.2 ± 0.86 | 0.97 ± 0.078 | 741.9 ± 37.0 | 2246 ± 134.7 | 29.0 ± 1.5 | 2836 ± 198.5 | 525.9 ± 42.0 | 61.5 ± 6.1 | 1.8 ± 0.19 | <1.6 | 19.6 ± 0.58 | 8.3 ± 0.74 |
| **S73** | 20.7 ± 0.83 | 1.3 ± 0.11 | 659.2 ± 65.9 | 2310 ± 115.5 | 28.0 ± 1.1 | 2891 ± 231.2 | 600.0 ± 41.9 | 22.9 ± 1.6 | 1.1 ± 0.074 | <1.6 | 15.7 ± 0.93 | 4.3 ± 0.38 |
| **S74** | 20.5± 2.5 | 1.3 ± 0.092 | 594.2 ± 29.7 | 2479 ± 272.6 | 29.0 ± 3.7 | 2904 ± 203.2 | 470.8 ± 32.9 | 27.2 ± 2.6 | 2.5 ± 0.19 | 169.5 ± 11.8 | 18.9 ± 1.5 | 4.9 ± 0.43 |
| **S75** | 23.0± 2.5 | 1.2 ± 0.097 | 638.8 ± 70.2 | 2335± 116.7 | 34.2 ± 2.7 | 2611 ± 130.5 | 480.0 ± 43.2 | 31.4 ± 0.62 | 0.93 ± 0.065 | 14.1 ± 0.98 | 21.5 ± 1.0 | 7.8 ± 0.85 |
| **S76** | 20.6 ± 2.7 | 1.4 ± 0.081 | 607.5 ± 30.3 | 2229 ± 89.1 | 29.7 ± 3.2 | 2506 ± 325.7 | 460.0± 45.9 | 9.7 ± 0.57 | 1.4 ± 0.069 | 14.0 ± 0.72 | 15.6 ± 1.7 | 5.0 ± 0.75 |
| **S77** | 22.8 ± 3.4 | 0.89 ± 0.061 | 578.8 ± 23.1 | 2202 ± 22.0 | 32.9 ± 1.9 | 2421 ± 145.2 | 560.0 ± 61.1 | 31.5 ± 1.8 | 0.71 ± 0.067 | <1.6 | 33.7 ± 1.3 | 3.3 ± 0.29 |
| **S78** | 15.1 ± 1.2 | 0.66 ± 0.059 | 653.1 ± 65.3 | 2277 ± 295.9 | 27.1 ± 2.4 | 1866 ± 111.9 | 470.3 ± 47.0 | 25.4 ± 2.5 | 3.4 ± 0.033 | <1.6 | 15.8 ± 1.1 | 5.7 ± 0.22 |
| **S79** | 35.7 ± 1.4 | 2.2 ± 0.23 | 522.6 ± 5.2 | 2585 ± 206.8 | 36.3 ± 2.1 | 3600 ± 252.0 | 499.1 ± 64.8 | 153.0 ± 7.6 | 3.7 ± 0.26 | 114.0 ± 9.1 | 39.4 ± 3.7 | 7.2 ± 0.28 |
| **S80** | 23.1 ± 1.4 | 1.5 ± 0.073 | 526.9 ± 68.5 | 2057 ± 123.3 | 30.3 ± 1.8 | 2576 ± 180.3 | 411.9 ± 28.8 | 18.2 ± 0.18 | 1.3 ± 0.077 | 23.3 ± 1.8 | 15.2 ± 1.0 | 5.1 ± 0.66 |
| **S81** | 22.6 ± 2.3 | 1.1 ± 0.10 | 363.4 ± 39.9 | 1583 ± 142.4 | 25.7 ± 1.8 | 2565 ± 51.3 | 390.1 ± 23.4 | 42.6 ± 4.6 | 1.5 ± 0.073 | <1.6 | 14.9 ± 1.1 | 4.7 ± 0.56 |
| **S82** | 21.6 ± 2.8 | 0.98 ± 0.058 | 460.2 ± 36.8 | 1757 ± 70.2 | 28.0 ± 2.2 | 2044 ± 20.4 | 371.9 ± 44.6 | 25.2 ± 1.7 | 2.7 ± 0.13 | <1.6 | 15.5 ± 1.3 | 5.5 ± 0.54 |
| **S83** | 22.7 ± 2.7 | 1.1 ± 0.054 | 567.4 ± 39.7 | 2464 ± 221.7 | 36.5 ± 2.5 | 2457 ± 245.6 | 470.7 ± 61.1 | 19.6 ± 2.3 | 2.7 ± 0.021 | 57.5 ± 3.4 | 17.9 ± 1.7 | 5.7 ± 0.27 |
| **S84** | 34.6 ± 2.8 | 0.99 ± 0.099 | 408.6 ± 24.5 | 1791 ± 107.5 | 38.5 ± 2.6 | 3103 ± 248.2 | 444.9 ± 66.7 | 11.55 ± 1.0 | 1.1 ± 0.063 | <1.6 | 36.6 ± 2.5 | 3.5 ± 0.14 |

**Table S4**. Estimated Pearson coefficients and p-values (in brackets) of elements, amino acids and fatty acids in DBSs (95% confidence interval)
